# Determination of local chromatin interactions using a combined CRISPR and peroxidase APEX2 system

**DOI:** 10.1093/nar/gkz134

**Published:** 2019-02-26

**Authors:** Wenqing Qiu, Zhijiao Xu, Min Zhang, Dandan Zhang, Hui Fan, Taotao Li, Qianfeng Wang, Peiru Liu, Zaihua Zhu, Duo Du, Minjia Tan, Bo Wen, Yun Liu

**Affiliations:** 1 MOE Key Laboratory of Metabolism and Molecular Medicine, Department of Biochemistry and Molecular Biology, School of Basic Medical Sciences, Zhongshan Hospital, Fudan University, Shanghai, China, 200032; 2 The Chemical Proteomics Center and State Key Laboratory of Drug Research, Shanghai Institute of Materia Medica, Chinese Academy of Sciences, Shanghai, China, 201203; 3 MOE Key Laboratory of Metabolism and Molecular Medicine, Institutes of Biomedical Sciences, and Department of Biochemistry and Molecular Biology, School of Basic Medical Sciences, Fudan University, Shanghai, China, 200032; 4 Division of Rheumatology, Huashan Hospital, Fudan University, Shanghai, China, 200040

## Abstract

The architecture and function of chromatin are largely regulated by local interacting molecules, such as transcription factors and noncoding RNAs. However, our understanding of these regulatory molecules at a given locus is limited because of technical difficulties. Here, we describe the use of Clustered Regularly Interspaced Short Palindromic Repeats and an engineered ascorbate peroxidase 2 (APEX2) system to investigate local chromatin interactions (CAPLOCUS). We showed that with specific small-guide RNA targets, CAPLOCUS could efficiently identify both repetitive genomic regions and single-copy genomic locus with high resolution. Genome-wide sequencing revealed known and potential long-range chromatin interactions for a specific single-copy locus. CAPLOCUS also identified telomere-associated RNAs. CAPLOCUS, followed by mass spectrometry, identified both known and novel telomere-associated proteins in their native states. Thus, CAPLOCUS may be a useful approach for studying local interacting molecules at any given chromosomal location.

## INTRODUCTION

In eukaryotic cells, DNA molecules are highly organized and tightly packed with repeating units of nucleosomes into chromatin. However, the chromatin architecture changes dynamically in living cells, so that local chromatin can be accessible to regulatory elements, such as transcription factors and noncoding RNAs (1). A number of mechanisms that regulate chromatin organization have been proposed in recent years (2). For example, each chromosome in the nucleus of a eukaryotic cell resides in a distinct region called a chromosome territory (3), which comprises many domains that are typically several megabases in size, termed topologically associating domains (TADs); within TADs, distal DNA elements dynamically interact with each other to regulate gene expression (4). Many factors, including CTCF, the cohesion complex and other DNA-binding proteins, are involved in the formation of TADs and the long-range interactions within them (5–7). In addition, epigenetic modifications, such as DNA methylation and histone modifications, and long noncoding RNAs play important roles in controlling gene expression by regulating the higher order structure of chromatin (8,9). These findings have brought us to an era of chromatin function research. However, a comprehensive understanding of chromatin function requires the identification of regulatory proteins and complexes that reside at a specific locus, which is challenging due to technical difficulties.

Numerous technologies have been proposed for studying local chromatin composition. For example, chromatin immunoprecipitation (ChIP) is a classic technique that is widely used to study the genome-wide distribution of a given protein. However, no method has been widely adopted to investigate local interacting molecules at a given genomic locus. Locked nucleic acid probes have been used to identify proteins bound to the telomeric region (10), but this approach is limited to highly repetitive regions of the genome. A LexA DNA-binding site was genetically incorporated into the yeast genome for site-specific chromatin purification (11); however, this method requires genomic engineering of the target genome, which can change the native environment of chromatin and is inefficient. Modified genome editing technologies such as transcription activator-like effector nucleases (TALEN) (12) and Clustered Regularly Interspaced Short Palindromic Repeats (CRISPR)-dCas9 (13,14) have been employed to enrich the desired genomic locus with catalytically inactive endonucleases. However, the TALEN-based approach requires that an amino acid sequence be designed for each locus, and CRISPR-based methods require that the cell be crosslinked with formaldehyde and that antibodies with high affinity and specificity are available. Moreover, these approaches cannot provide functional analyses of native chromatin or genome-wide specificity.

Here, we describe a method named CAPLOCUS (Combining CRISPR and peroxidase APEX2 system to identify local chromatin interactions) to investigate local interactions for a given genomic locus. We validated our system by capturing human telomeres, a repetitive region on chromosome 13, and two single-copy loci on chromosome 11. Genome-wide sequencing revealed efficient enrichment of the target regions as well as genomic regions with long-range interactions. CAPLOCUS also identified telomere-associated RNAs. The combination of CAPLOCUS with mass spectrometry (MS) allowed us to identify many known and unknown telomere-associated proteins. Hence, CAPLOCUS provides a new approach for investigating local interacting molecules at any given chromosomal location.

## MATERIALS AND METHODS

### Plasmids

Addgene plasmid 64107 was used to express dCas9. To create the MS2-APEX2_NLS fusion protein expression vector, APEX2 was amplified by polymerase chain reaction (PCR) from pcDNA3 Connexin43-GFP-APEX2 (Addgene plasmid: 49385) and cloned into the pHAGE-EFS-MCP-3XBFPnls vector (Addgene plasmid: 75384) with BamHI and XhoI. The small-guided RNA (sgRNA) expression vectors were cloned by inserting the annealed oligos into pLH-sgRNA1-2XMS2 (Addgene plasmid: 75389) at the BbsI site. All sgRNA sequences are shown in Supplementary Table S1.

### Cell culture

Human embryonic kidney HEK293T cells were cultured at 37°C under 5% CO_2_ in high-glucose Dulbecco’ Modified Eagle’s Medium (Life Technologies, Carlsbad, CA, USA) supplemented with 10% fetal bovine serum (FBS; Sigma, St. Louis, MO, USA), 1% penicillin/streptomycin (Life Technologies), and passaged at 1:5 every 2 days. K562 cells were cultured at 37°C under 5% CO2 in RPMI 1640 medium supplemented with 10% FBS and 1% penicillin/streptomycin. Mycoplasma testing was performed each week.

### Imaging of human telomeres

HEK293T cells were transfected with MS2-BFP_NLS and telomere-specific sgRNA (sgTelomere) or negative control sgRNA (sgGal4) in a 6-well chambered coverglass. The distribution of MS2-BFP_NLS was determined on a fluorescence microscope (Leica SP5) with 63× objective lens.

### Proximity labeling

Proximity labeling was performed as described by Hung *et al.* (15). Briefly, cells were transiently transfected with polyethylenimine (Polysciences Inc., Warrington, PA, USA) according to the manufacturer’s protocols. A total of 900 ng of dCas9 plasmid DNA, 4.5 μg sgRNA plasmid DNA and 120 ng MS2-APEX2_NLS plasmid DNA were co-transfected in cells at 60–80% confluence in T75 flask. Twenty-four hours after transfection, cells were treated with 500 μM biotin-phenol (Iris Biotech GmbH, Germany) for 30 min, after which hydrogen peroxide was added to a final concentration of 1 mM and the cells were incubated for 1 min. Then, the reaction was immediately quenched by addition of quench buffer (10 mM sodium azide, 10 mM sodium ascorbate and 5 mM Trolox). Finally, the cells were either harvested directly or fixed in formaldehyde.

### Affinity purification of biotinylated protein–DNA complexes and high-throughput sequencing

A total of 2 × 10^7^ cells were transfected with target sgRNA or the sgGal4 control. After proximity labeling, cells were fixed in 1% formaldehyde and incubated at room temperature for 10 min. Glycine was added to a final concentration of 125 mM to terminate the reaction for 5 min at room temperature. After lysed in 1 ml hypotonic buffer (20 mM 4-(2-hydroxyethyl)-1-piperazineethanesulfonic acid (HEPES) (pH 7.5), 10 mM potassium chloride, 1 mM ethylenediaminetetraacetic acid (EDTA), 0.1 mM activated sodium orthovanadate, 0.2% nonyl phenoxypolyethoxylethanal (NP-40), 10% glycerol and protease inhibitor cocktail) for 15 min, cell lysates were centrifuged at 13 000 *g* for 1 min at 4°C. The chromatin pellet was resuspended in 500 μl ChIP dilution buffer (20 mM Tris–HCl (pH 8.0), 2 mM EDTA, 150 mM NaCl, 0.1% sodium dodecyl sulphate (SDS), 1% Triton X-100 and protease inhibitor cocktail) and sonicated to yield fragments with an average length of 100–500 bp on the Bioruptor Pico sonication device. Fragmented chromatin was centrifuged at 14 000 *g* for 15 min at 4°C, after which 5% of the supernatant was saved as the input sample and stored at −20°C. Sheared chromatin was pre-cleared for 1 h at 4°C using 50 μl Protein A, and then the supernatant was incubated with 50 μl M-280 Streptavidin Dynabeads (Life Technologies) overnight at 4°C with rotation. Then, the Dynabeads were washed sequentially with 2% SDS, high-salt buffer (50 mM HEPES (pH 7.5), 500 mM NaCl, 1 mM EDTA, 0.1% sodium deoxycholate, 1% Triton X-100), LiCl buffer (10 mM Tris–HCl (pH 8.0), 250 mM LiCl, 1 mM EDTA, 0.5% NP-40, 0.5% sodium deoxycholate) and Tris-EDTA (TE) buffer. To reverse crosslinking, the beads and input samples were incubated with SDS elution buffer (50 mM Tris–HCl (pH 8.0), 10 mM EDTA, 1% SDS) and incubated at 70°C overnight in a water bath. After Proteinase K and RNase A treatment, DNA was purified using the QIAquick PCR Purification Kit (Qiagen, Hilden, Germany) and used as a template for quantitative PCR (qPCR) to determine the fold enrichment of each target. DNA libraries were prepared using the Ultra II DNA Library Prep Kit for Illumina (NEB) and sequenced on the Hiseq X Ten platform according to the manufacturer’s instructions. The sequencing depth for each library was between 11.74 and 35.83 million reads. The clean reads were mapped to the UCSC hg19 Human Genome using bwa aln algorithm (16) with default parameters and only uniquely mapped reads were kept for downstream analysis. PCR duplications were removed using samtools rmdup, and MACS2 (17) was applied for each sample for peak calling with the ‘-g hs -f BAMPE -B’ parameter. None of these called peaks appeared in the blacklists of the ENCODE project (18). The peaks overlapping between samples with sgRNA targeting C11 or C13 and the sgGal4 negative controls were removed with bedtools. For experiments with replications, only peaks shared between two replicates were kept. We identified the target sites and potential interaction loci from two replicates for final visualization with IGV (19).

### **Transposase-accessible chromatin with sequencing** (**ATAC-Seq)**

The ATAC-Seq libraries were prepared as described previously (20). Briefly, 5 × 10^4^ cells were lysed in lysis buffer (10 mM Tris–HCl (pH 7.4), 10 mM NaCl, 3 mM MgCl_2_ and 0.1% Igepal CA-630) for 20 min on ice, followed by centrifugation at 500 *g* for 10 min to isolate nuclei. Tn5 transposase and fragment buffer was added and incubated at 37°C for 30 min. The fragmented DNA was then purified using the Qiagen MinElute PCR Purification Kit. PCR was performed to amplify purified transposed DNA fragments for 13 cycles with the following conditions: 72°C for 5 min; 98°C for 30 s; thermocycling at 98°C for 10 s, 63°C for 30 s and 72°C for 1 min. Amplified libraries were purified with 0.8× AMPure beads and sequenced on the Illumina Hiseq X Ten platform. Sequencing raw reads were mapped to the UCSC hg19 Human Genome using Bowtie2 with -X 2000. Uniquely mapped reads were used for duplication removal and then for nucleosome positioning and peak calling. The positions of nucleosomes were inferred from the distribution of library insert size, which was identified by picard tools. We converted the alignment files to bedpe format using bedtools, offset the reads aligning to the + strand by +4 bp and the reads aligning to the - strand by −5 bp, and then applied MACS2 to identify ATAC-Seq peaks with the ‘-t ATAC -g hs -f BEDPE -B’ parameter.

### Chromatin immunoprecipitation followed by sequencing (ChIP-Seq)

A total of 1 × 10^7^ cells were used for ChIP-Seq experiments. Cells were first crosslinked with 1% formaldehyde for 10 min, then quenched by adding a final concentration of 125 mM glycine. Cells were lysed in 1 ml hypotonic buffer followed by centrifugation at 13 000 *g* for 1 min at 4°C. The chromatin pellets were resuspended in 500 μl ChIP dilution buffer and sonicated to yield DNA fragments with an average length of 100–500 bp. Sheared chromatin was pre-cleared with 50 ul Protein G Dynabeads for 1 h at 4°C, followed by incubation with 4 ug of anti-Cas9 antibody (ab191468; Abcam), together with 50 μl of Protein G Dynabeads overnight at 4°C with rotation. The Dynabeads were then washed sequentially with 2% SDS, high-salt buffer, LiCl buffer and TE buffer as described above. After reverse crosslinking, DNA was treated with Proteinase K and RNase A and purified with QIAquick PCR Purification Kit (Qiagen, Hilden, Germany). DNA libraries were constructed and sequenced as described above.

### Quantitative PCR (qPCR)

qPCR was performed using the LightCycler 480 SYBR Green I Master Mix (Roche). The relative enrichment of amplified DNA fragments was determined relative to input DNA and normalized to that of a negative control region (SOX2) to eliminate background in cells transfected with sgTarget, and then normalized to that in cells transfected with sgGAL4.

\begin{eqnarray*}&&{\rm{Relative\ }}\,{\rm{enrichment}} \nonumber\\ &&= \frac{{{\rm{Target}}\left( {\frac{{{\rm{pulldow}}{{\rm{n}}_{{\rm{sgTarget}}}}}}{{{\rm{inpu}}{{\rm{t}}_{{\rm{sgTarget}}}}}}} \right)}}{{{\rm{SOX}}2\left( {\frac{{{\rm{pulldow}}{{\rm{n}}_{{\rm{sgTarget}}}}}}{{{\rm{inpu}}{{\rm{t}}_{{\rm{sgTarget}}}}}}} \right)}}/\frac{{{\rm{Target}}\left( {\frac{{{\rm{pulldow}}{{\rm{n}}_{{\rm{sgGAL}}4}}}}{{{\rm{inpu}}{{\rm{t}}_{{\rm{sgGAL}}4}}}}} \right)}}{{{\rm{SOX2}}\left( {\frac{{{\rm{pulldow}}{{\rm{n}}_{{\rm{sgGAL}}4}}}}{{{\rm{inpu}}{{\rm{t}}_{{\rm{sgGAL}}4}}}}} \right)}}\end{eqnarray*}

A standard curve was conducted for each assay. Reactions were carried out in triplicates. PCR primers are provided in the Supplementary Table S1.

### Circular chromosome conformation capture with sequencing (4C-Seq)

4C library was prepared as described previously with minor modifications (21). Briefly, a total of 1 × 10^7^ HEK293T cells were crosslinked for 10 min at room temperature with 2% formaldehyde, then quenched by adding a final concentration of 125 mM glycine. Cell pellets were lysed in 5 ml cold lysis buffer (50 mM Tris–HCl (pH 7.5), 150 mM NaCl, 5 mM EDTA, 0.5% NP-40, 1% Triton X-100 and protease inhibitor cocktail). The nuclei pellet was resuspended in 0.5 ml of 1.2 × restriction buffer, placed at 37°C, and 15 μl 10% SDS (final concentration 0.3%) was added and the tubes were incubated for 1 h at 37°C while shaking at 900 rpm, followed by the addition of 75 μl of 20% Triton X-100 and incubated for 1 h at 37°C while shaking at 900 rpm. 400 U of DpnII was added to the sample and the sample was incubated overnight at 37°C while shaking at 900 rpm. The enzyme was inactivated by adding SDS at a final concentration of 1.6% and incubated for 20 min at 65°C. The digested nuclei were diluted with 6.125 ml of 1.15 × T4 ligation buffer supplemented with 375 μl of 20% Triton X-100 (final concentration 1%). After incubation at 37°C for 1 h, nuclei were ligated by adding 50U T4 DNA ligase with rotation overnight at 16°C. DNA crosslinks were reversed by adding 30 μl proteinase K (10 mg/ml) and digested at 65°C overnight. Then 30 μl RNase A (10 mg/ml) was added and the tube was incubated for 45 min at 37°C. The DNA was purified by phenol extraction and ethanol precipitation and dissolved in 150 μl of 10 mM Tris–HCl (pH 7.5). To trim the large circles, a second digestion and ligation was performed. To 150 μl purified DNA, 50 U Taq I enzyme was added and digested overnight at 37°C. Then, ligation was carried out with 100 U of ligase in 14 ml total volume at 16°C overnight. DNA was purified by ethanol precipitation and dissolved in 150 μl of 10 mM Tris–HCl (pH 7.5). 4C PCR was performed with a total amount of input of 3.2 μg and separated into 16 reactions. PCR products were pooled and purified using Qiagen PCR cleanup kit. The DNA was eluted in 10 mM Tris–HCl (pH 7.5) and sequenced on the Illumina Hiseq X Ten platform. PCR primers are provided in the Supplementary Table S1.

### 4C-Seq data analysis and comparison of chromatin interactions with CAPLOCUS

4C-Seq data analysis was performed using 4C-ker package (22) according to the software’s instructions. Briefly, 4C-Seq reads were mapped to a reduced human genome (hg19) consisting of unique sequence fragments adjacent to the primary restriction enzyme sites, which was created by a script in the package. Then a counts profile was created from mapped data, and was used to analyze 4C interactions both cis and trans. The low and high interaction signals identified by 4C-ker were both retained. The interaction profile was visualized by a circular plot created by Circos (23). The comparison with CAPLOCUS was performed using GenomicRanges (24).

### Affinity purification of biotinylated protein–RNA complexes and RNA library preparation

Assays were performed as described above with some modifications. In brief, after proximity labeling, cells were fixed in 0.2% formaldehyde. Fixed cell pellet was lysed in 1 ml hypotonic buffer supplemented with 100 U/ml RNasin and 0.5 mM dithiothreitol (DTT). The chromatin pellet was resuspended in 500 μl RIPA buffer (50 mM Tris–HCl (pH 8.0), 5 mM EDTA, 150 mM NaCl, 0.1% SDS, 0.5% sodium deoxycholate, 1% Triton X-100, 100 U/ml RNasin, 0.5 mM DTT and protease inhibitor cocktail) and sheared to yield fragments with an average length of 100–1000 bp. The sheared samples were centrifuged at 14 000 *g* for 15 min at 4°C, after which 5% of the supernatant was saved as the input sample and stored at −80°C. The remaining supernatant was transferred to a new RNase free microfuge tube and incubated with 50 μl M-280 Streptavidin Dynabeads that had been equilibrated by two washes in RIPA buffer with RNasin at 4°C overnight. Beads were washed sequentially with 1 ml buffer below at 4°C for 10 min with rotation: RIPA buffer, high salt buffer (50 mM Tris–HCl (pH 8.0), 1 M NaCl, 0.1% SDS, 1% TritonX-100, 5 mM EDTA, 100 U/ml RNasin, 0.5 mM DTT and protease inhibitor cocktail), urea buffer (2 M urea, 50 mM Tris–HCl (pH 8.0), 0.1% SDS, 1% TritonX-100, 5 mM EDTA, 100 U/ml RNasin, 0.5 mM DTT and protease inhibitor cocktail), RIPA buffer and TE buffer. To reverse crosslinking, the beads and input samples were incubated with 100 μl SDS elution buffer supplemented with 2 μl proteinase K (20 mg/ml) and 20 U RNasin at 50°C for 1 h, followed by at 65°C for 1.5 h. Eluted samples were transferred to a new RNase free microfuge tube and DEPC water was added to reach 250 μl. An amount of 750 μl Trizol (Life Technologies) was added to the samples and RNA was isolated following manufacturer’s protocol. For quantitative real-time-PCR analysis, RNA was reverse transcribed by PrimeScript™ RT reagent Kit with gDNA Eraser (TAKARA). PCR primers are provided in the Supplementary Table S1. For library construction, 50–150 ng RNA was first treated with DNase I, then converted to complementary DNA by using PrimeScript II RTase (TAKARA). Thereafter, libraries were prepared using NEBNext Ultra Directional RNA Library Prep Kit (NEB) and sequenced on the Hiseq X Ten platform according to the manufacturer’s instructions.

### CAPLOCUS followed by RNA-Seq data analysis and comparison with MARGI (mapping RNA–genome interactions)

Kallisto 0.42.5 (25) was used for expression level quantification with default parameters, and clean reads were pseudoaligned to the human GENCODE v24 reference transcriptome. Estimated read counts of each transcript were used to calculate the fold enrichment between sgTelomere and sgGAL4. RNAs with fold enrichment ≥3 in sgTelomere versus sgGAL4 was defined as enriched. Reads aligning to ribosomal RNA were removed from further analysis. For telomeric repeat-containing RNA (TERRA) analysis, reads containing (TTAGGG)_4_ or (CCCTAA)_4_ segment were counted, and then the number of these reads were divided by the total number of clean reads to assess the enrichment of TERRA for each sample. To compare with mapping RNA–genome interactions (MARGI) (26), RNA–DNA interactions from HEK293T cells detected by pxMARGI was analyzed. RNAs associated with the telomeric region were retrieved and compared with CAPLOCUS.

### Streptavidin pull-down of biotinylated proteins and western blot analysis

Streptavidin pull-down of biotinylated proteins was performed as previously described (15) with minor modifications. Briefly, 4 × 10^7^ cells were transfected and proximity-labeled as described above and harvested directly after quenching. Then, the cell pellets were resuspended in hypotonic buffer and lysed for 15 min. After centrifugation at 13 000 *g* for 10 min at 4°C, the pellets were resuspended in cold lysis buffer (50 mM Tris, 150 mM NaCl, 0.2% NP-40, 5% glycerol, 1.5 mM MgCl_2_) and sonicated (Diagenode Bioruptor, High, 10 min, 30 s on, 30 s off). Cell extracts were clarified by centrifugation, and the amount of protein in each supernatant was measured; 1% of the supernatant was saved as input for western blot analysis. Supernatant (1 mg) was incubated with 50 μl M-280 Streptavidin Dynabeads (Life Technologies) overnight at 4°C with rotation. Then, the beads were collected on a magnetic plate and washed with SDS wash buffer, high-salt buffer, LiCl buffer and TE buffer as described above. The beads were resuspended in 30 μl of 3 x protein loading buffer supplemented with 2 mM biotin and 20 mM DTT and boiled for 10 min. Eluted biotinylated proteins were subjected to 10% sodium dodecylsulphate-polyacrylamide gel electrophoresis (SDS-PAGE) followed by western blot analysis. The antibodies used in this study were streptavidin-HRP (21130; Life Technologies), anti-Cas9 (ab191468; Abcam, Cambridge, UK), anti-TRF1 (ab10579; Abcam), anti-TRF2 (sc-271710; Santa Cruz Biotechnology, Dallas, TX, USA), anti-RAP1 (sc-53434; Santa Cruz Biotechnology) and anti-POT1 (ab124784; Abcam).

### **Stable isotope labeling by amino acids** (**SILAC)**

The two-state SILAC was performed as described by Hung *et al.* (15). Briefly, HEK293T cells were seeded into two T25 flasks and cultured in SILAC media to metabolically incorporate heavy or light isotopes of lysine and arginine into their proteomes. After 8–10 days, the labeling efficiency was evaluated. Cells were expanded into two T75 flasks per condition, and transfected with dCas9 plasmid, MS2-APEX2_NLS, and the sgRNA of interest for the heavy state and dCas9, MS2-APEX2_NLS, and sgRNA Gal4 for the light state. Twenty-four hours after transfection, heavy and light state cells were separately labeled with biotin. Streptavidin-mediated pull down of biotinylated proteins was performed as described above with minor modifications. After protein quantification, 2 mg protein per state was mixed and incubated with 100 μl M-280 Streptavidin Dynabeads (Life Technologies) overnight at 4°C with rotation. The eluted biotinylated proteins were subjected to SDS-PAGE followed by reduction, an alkylation reaction, and in-gel trypsin digestion. The tryptic peptides were extracted and dried in a SpeedVac for high-performance liquid chromatography tandem mass spectrometry (HPLC-MS/MS) analysis.

### HPLC-MS/MS

The digested peptides were dissolved in buffer A (0.1% formic acid in water, v/v). Samples were analyzed on an Orbitrap Fusion Mass Spectrometer (Thermo Scientific, Waltham, MA, USA) and eluted for 60 min with a gradient of 8 to 45% acetonitrile using a MS2-based method. Full-time scans were performed with the maximum injection time of 200 ms and automatic gain control (AGC) targets of 1 million ion counts, followed by an MS/ MS scan with a maximum injection time of 50 ms and AGC targets of 5000 ion counts. Full MS spectrum scans (m/z 450–1500) were performed at a resolution of 120 000 at 400 m/z, followed by MS/MS fragmentation of 3 s top speed with high collision dissociation energy of 32%.

### MS data analysis

The MS data were analyzed by MaxQuant software (Version 1.4.1.2) against the Uniprot human databases. The search was performed with the following parameters: carbomidomethyl (C) as the fixed modification and acetylation (protein N-term), Label: 13C(6) (K), Label: 13C(6)15N(4) (R), Oxidation (M), biotinylation by biotin-phenol (Y; (C18H23N3O3S)) as variable modifications. Other parameters were as follows: two missed trypsin cleavages were permitted, the error of precursor mass tolerance was 10 ppm with fragment ion as 0.5 Da, the peptide false discovery rate was 0.01, and a minimum peptide length of six was required. After analyzed by MaxQuant, the un-normalized *H/L* SILAC ratio provided by MaxQuant were used to normalized the *H/L* ratio manually as described by Hung *et al.* (15). A list of proteins known to reside in cytoplasm and should not be biotin-labeled were chose as false positives (Supplementary Table S2). For normalization, the median of the false positive proteins was first calculated, and then the ratio of each detected protein was divided by this value. We surveyed the distribution of the false positive proteins, and observed that 83% of these proteins displayed *H/L* ratio <1.5-fold (Supplementary Figure S6A), thus proteins with *H/L* ratio ≥1.5 were considered to be enriched in sample with sgTelomere relative to the sgGal4 control. Identified proteins were subject to functional profile analysis for gene clusters using R package clusterProfiler v.3.6.0 (27) with a focus on Reactome pathway with default parameters (adjusted *P*-value < 0.05; p.adjust method = ‘BH’).

## RESULTS

### CAPLOCUS

**Figure 1.**
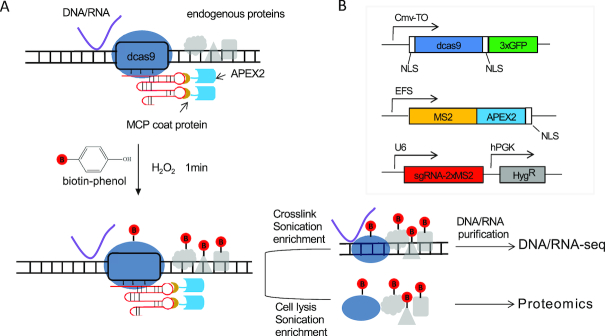
Combining CRISPR system with peroxidase APEX2 to identify local chromatin interactions. (**A**) Schematic of CAPLOCUS system. (**B**) The three components of the CAPLOCUS system: TO-NLS_dCas9_NLS, the RNA binding partner fused with peroxidase APEX2_NLS, and target-specific sgRNA.

To identify molecules (e.g. DNAs, RNAs and proteins) at a specific genomic location, we developed a method based on a modified CRISPR system (Figure 1A). Optimized sgRNA with two MS2 RNA elements was also used (28). The MS2 coat protein was fused to an engineered ascorbate peroxidase called APEX2 (29), which is capable of in-cell biotin labeling of nearby proteins in the presence of substrates, biotin-phenol and hydrogen peroxide (Figure 1B). The efficiency of labeling with the fused MS2-APEX2_NLS protein was verified by western blotting (Supplementary Figure S1). After transforming the cells with specific sgRNAs followed by *in vivo* biotinylation, the cells underwent two different treatments to enrich DNA/RNA molecules or proteins. For enrichment of DNA/RNA, the cells were crosslinked and sonicated before streptavidin affinity purification followed by sequencing. For protein enrichment, the cells were lysed and the biotin-labeled proteins were enriched with streptavidin affinity purification followed by detection by western blotting or MS. We believe that several advantages can be achieved with this approach. First, APEX2 catalyzes one-electron oxidations to rapidly produce short-lived (<1 ms) biotin-phenoxyl radicals with a 20-nm labeling radius (15), enabling temporal labeling and identification of local interacting molecules at a given time point. Second, compared to antibody-based affinity strategies (13,14), the robust streptavidin-biotin interaction can endure more rigorous handling, leading to high specificity with limited contamination. Third, because proximity labeling occurs directly on nearby proteins for a given locus, native chromatin can be used for protein enrichment without the need to fix the cells, thereby providing more accurate results.

### Evaluation of the efficiency and specificity of CAPLOCUS

**Figure 2.**
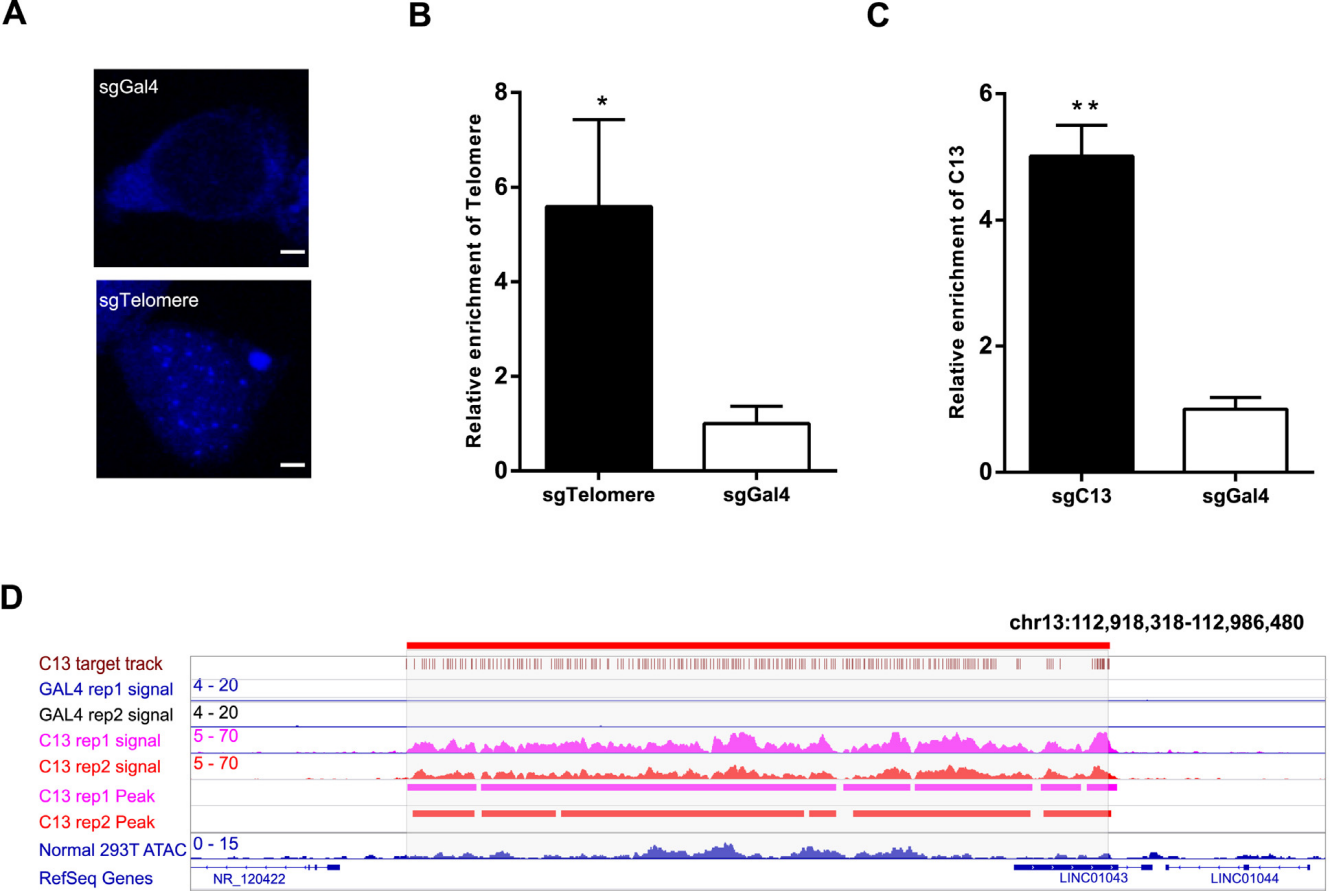
CAPLOCUS can efficiently isolate repetitive regions. (**A**) Labeling of human telomeres in HEK293T cells following co-expression of dCas9, the RNA-binding partners MS2 fused with blue fluorescent protein (BFP) and the indicated sgRNA; scale bar, 2 μm. (**B**) Isolation of telomere region by CAPLOCUS. qPCR analysis shows significant enrichment of telomere DNA. The relative enrichment was determined relative to input DNA and normalized to that of a negative control region (SOX2), and then normalized to that in cells transfected with sgGAL4. Error bars are mean ± SEM of three experiments and analyzed by a two-sided *t-*test, *P < 0.05, **P < 0.01. (**C**) Isolation of a repeat region on chromosome 13: 112930173 - 112968847 (C13). qPCR analysis shows significant enrichment of this region. The relative enrichment was determined as described in (B). Error bars are mean ± SEM of three experiments and analyzed by a two-sided *t*-test, *P < 0.05, **P < 0.01. (**D**) CAPLOCUS on the C13 region followed by the next generation sequencing. The replicate experiments (rep1 and rep2) are shown. Cells expressing sgGal4 were analyzed as negative controls. The region encompassing the C13 locus is indicated in red bars on the top. The dCas9/sgC13 binding locations are indicated in brown. The peaks called in the replicate experiments are shown.

As proof of concept, we first utilized a telomere region to evaluate the efficiency of CAPLOCUS. Telomeres are specialized regions at both ends of a chromosome that consist of repetitive DNA sequences of up to 5–15 kb, which allows the recruitment of multiple MS2-APEX2_NLS proteins using a single sgRNA. We used a previously validated sgRNA to target telomeres (28). HEK293T cells transfected with the MS2-BFP_NLS fusion protein showed fluorescent puncta in cells expressing sgRNAs targeting telomeres (sgTelomere); in contrast, no obvious puncta were found in cells without sgRNAs targeting telomeres (sgGal4) (Figure 2A). This suggests that MS2 fusion proteins can be successfully recruited to targeted loci via binding to two MS2 RNA elements on the sgRNA. To determine if CAPLOCUS could enrich a target region on chromatin, HEK293T cells were transfected with plasmids expressing CRISPR/dCas9, the MS2-APEX2_NLS fusion protein and the sgTelomere or sgGal4 followed by crosslinking, proximity labeling and purification. qPCR showed a significant enrichment (5.58-fold) of the telomere region compared to the sgGal4 control (Figure 2B), indicating efficient purification of the targeted chromatin. Next, we tested this approach in another repetitive region located on chromosome 13 (chr13: 112,930,173-112,968,847) (named C13) and observed a similar 5-fold enrichment compared to the control (Figure 2C). To determine the specificity and reproducibility of CAPLOCUS at a genome-wide level, we performed CAPLOCUS for the C13 region and subjected the DNA samples to next-generation sequencing. As expected, the targeted C13 region was highly enriched compared with other genomic regions, whereas the sgGal4 control showed no enrichment in this region (Figure 2D). A biological replication showed a similar result (Figure 2D), suggesting that CAPLOCUS is highly reproducible. Even though the target C13 region was enriched, we did notice that the level of enrichment differed within the region. To investigate whether this difference in enrichment is related to dCas9/sgC13-binding sites, we called the peaks from both experiments and noticed that, for three regions which did not contain peaks in both experiments, there were no dCas9/sgC13-binding sites (Supplementary Figure S2A). Additionally, most of other regions with weak signals correspond to regions with no or low density of dCas9/sgC13-binding sites (Supplementary Figure S2A), suggesting a high specificity of CAPLOCUS. Furthermore, by comparing the results from two replicates, we observed highly specific enrichment of the target region (Supplementary Figure S2B), further indicating the high specificity of the method.

To evaluate whether the chromatin accessibility affects the performance of CAPLOCUS, we did ATAC-Seq experiment on HEK293T cells. While there is difference for the chromatin accessibility in C13, this difference showed no correlations with the enrichment of the target region, suggesting the chromatin accessibility does not affect the CAPLOCUS system (Figure 2D). Moreover, ATAC-Seq experiments on cells transfected with dCas9/sgC13 or dCas9/sgCAL4 showed similar distribution of nucleosome positioning (Supplementary Figure S2C) as well as local peak patterns compared to un-transfected HEK293T cells (Supplementary Figure S2D), indicating the CAPLOCUS system does not affect both genome-wide and local chromatin accessibility.

**Figure 3.**
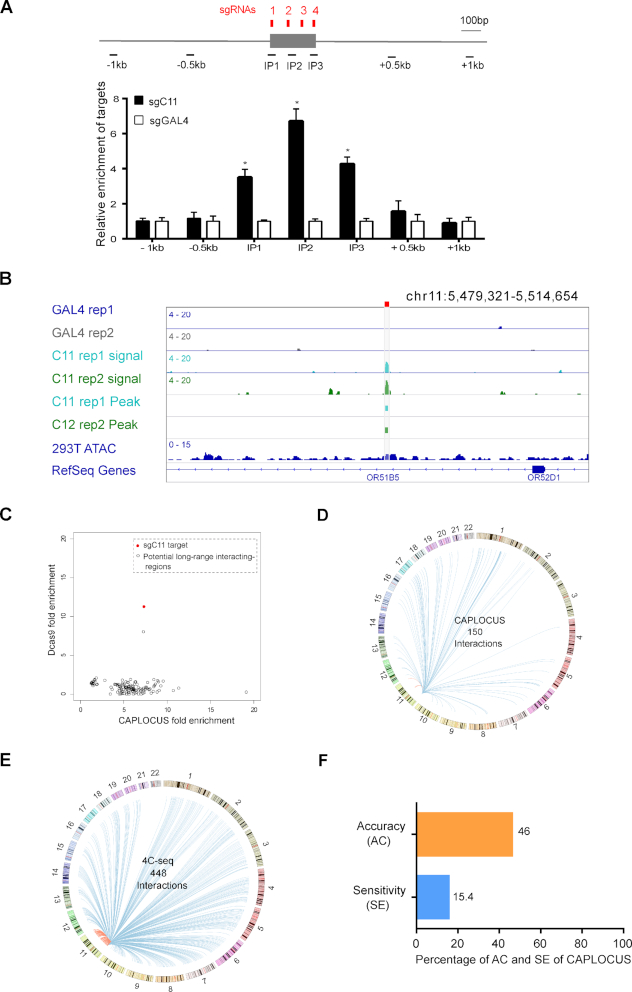
CAPLOCUS can identify long-range interactions on a single-copy locus. (**A**) A diagram depicting the target region on chromosome 11: 5497550–5498040 (C11) and the flanking sequence is shown on the top. C11 sgRNA targeting locations are indicated in red and qPCR amplicon regions are indicated in black. qPCR analysis shows significant enrichment of C11 region in cells expressing sgC11 in comparison to those with sgGal4. No significant enrichment is observed 500 or 1000 bp up- and downstream of the C11 target. The relative enrichment was calculated as described in Figure 2B. Error bars are mean ± SEM of three experiments and analyzed by a two-sided *t-*test, *P < 0.05. (**B**) The C11 target region is directly captured with CAPLOCUS followed by the next generation sequencing. Two replicates are shown. Cells expressing sgGal4 were analyzed as negative controls. The sgC11 target region is indicated as red bar on the top. (**C**) Comparison of CAPLOCUS and dCas9 ChIP-Seq results on the chromatin regions enriched by CAPLOCUS targeting the C11 target. The data point for the C11 target region is shown in red. Most of the other regions were only enriched by CAPLOCUS, but not by dCas9 ChIP-Seq, excluding the possibility of dCas9 off-target bindings. (**D**) Circle plot of the long-range interactions at C11 identified by CAPLOCUS. The identified inter- and intra-chromosomal interactions are shown as red and blue, respectively. (**E**) Circle plot of the long-range interactions at C11 identified by 4C-Seq. The identified inter- and intra-chromosomal interactions are shown as red and blue, respectively. (**F**) Sensitivity (SE) and accuracy (AC) of CAPLOCUS in comparison to 4C-Seq. Among the interactions identified by CAPLOCUS, 46% of them are found in 4C-Seq. (SE = the number of overlapping interactions between CAPLOCUS and 4C-Seq / the total number interactions from 4C-Seq; AC = the number of overlapping interactions between CAPLOCUS and 4C-Seq / the total number of interactions from CAPLOCUS)

To test the efficiency and specificity of CAPLOCUS for a single-copy locus, we chose a 233-base pair (bp) region on chromosome 11 (chr11: 5,497,611-5,497,843) (named C11), which is a genomic region with high chromatin accessibility marked by DNase I hypersensitive sites. Four sgRNAs were designed to target this region and were transfected together to enrich C11. Similar to the results from repetitive regions, the C11 region was enriched in an sgRNA-dependent manner. Moreover, no significant enrichment was observed for nearby chromatin regions 0.5 kb up- or downstream of the sgRNA-targeted C11 region (Figure 3A), suggesting that CAPLOCUS can specifically capture a targeted single-copy locus with high specificity and high resolution.

### CAPLOCUS can identify long-range DNA interactions

Long-range DNA interactions play important roles in gene regulation and activity. Several approaches, such as chromosome conformation capture techniques (3C) (30), 4C (31), 5C (32), Hi-C (33) and chromatin interaction analysis by paired-end tag (ChIA-PET) (34), have been utilized to identify genome-wide chromatin interactions. To evaluate whether CAPLOCUS can be used to identify long-range DNA interactions at a single-copy locus, DNA samples enriched by CAPLOCUS that targeted the C11 region were subjected to next-generation sequencing. As demonstrated in Figure 3B, the C11 target region was successfully enriched compared to the controls. Additionally, a distant region (chr11: 465,8100 - 4658,849) previously reported to interact with C11 in HEK293T cells using the 3C method (35) (Supplementary Figure S3) was also confirmed by CAPLOCUS. Genome-wide analysis of called peaks showed a specific enrichment of C11 as well as some additional sites, indicating potential long-range chromatin interactions (Supplementary Figure S4A). To exclude the possibility that these interactions are result of off-target binding by dCas9/sgC11 (36,37), ChIP-Seq was performed by using antibody against dCas9. While the target region was enriched in both CAPLOCUS and dCas9 ChIP-Seq (the red dot in Figure 3C and Supplementary Figure S4B), there are many other sites that were only captured by the CAPLOCUS system, but not by dCas9 ChIP-Seq (Figure 3C), suggesting that these potential chromatin interaction regions were not a result of dCas9/sgC11 off-target binding. To validate these potential long-range interactions, we performed additional independent CAPLOCUS followed by qRT-PCR for one of these regions, which is specifically captured by CAPLOCUS (Supplementary Figure S4C) and observed significant enrichment in this interacting region compared to the negative control (Supplementary Figure S4D), indicating that CAPLOCUS is capable of identifying long-range DNA interactions for a given single-copy locus.

We next aim to compare the long-range DNA interactions identified by CAPLOCUS with the interactions derived from circular chromosome conformation capture (4C) (31). 4C assay was performed in HEK293T cells to enrich genomic regions interacting with a 993-bp region (chr11:5497151-5498143) encompassing the C11 target. In total, 448 distal interactions were obtained from the 4C-Seq, compared to 150 interactions from CAPLOCUS analysis (Figure 3D and E). Among them, 46% of the interactions identified by CAPLOCUS can be found in 4C-Seq (Figure 3F), including the interactions verified above by qPCR, implying high accuracy of CAPLOCUS and general agreement between the two methods. To test the generality of CAPLOCUS, we applied the method in another single-copy locus in a different cell line. The β-globin locus is a well-characterized region with β-globin locus control region and downstream hemoglobin genes flanked by HS5 and 3′HS1 sites (38,39). Previous studies using 3C (40), UMI-4C (41) and CTCF ChIA-PET (42) showed that the HS5 site interacted with 3′HS1 site through long-range chromatin interaction in K562 cells (Supplementary Figure S5A). To replicate this, CAPLOCUS was performed in K562 cells with two sgRNAs designed to target the 3′HS1 site. qPCR showed a significant enrichment (6.26-fold) of the 3′HS1 region compared to the sgGal4 control, and no enrichment was observed for nearby chromatin regions 0.5 kb up- or downstream of the 3′HS1 target site (Supplementary Figure S5B). The interacting HS5 site was also evaluated and a significant 2-fold enrichment was observed compared to the sgGAL4 control (Supplementary Figure S5B). In comparison, 5C (43) or *in situ* Hi-C (44), which can indicate higher levels of chromatin structures including compartments and TADs, failed to identify this interaction (Supplementary Figure S5A). Thus, this shows that CAPLOCUS provides an attractive alternative to 3C or 4C approaches, even though it does not require proximity ligation and enzyme digestion, and yields high-resolution data.

### CAPLOCUS can identify telomere-associated RNA

**Figure 4.**
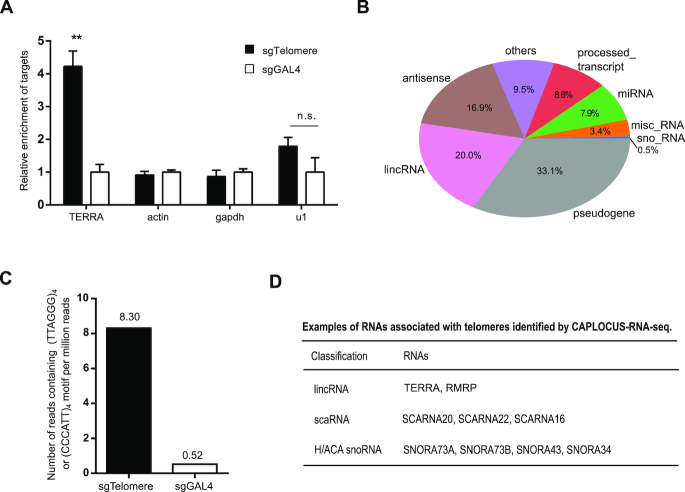
CAPLOCUS can identify telomere-associated RNAs. (**A**) qRT-PCR analysis shows significant enrichment of TERRA by CAPLOCUS followed by reverse transcription. The relative enrichment was determined relative to input, and normalized to that in cells transfected with sgGAL4. The enrichment of negative controls, actin, gapdh and u1, were also shown. Error bars are mean ± SEM of three experiments and analyzed by a two-sided *t-*test, **P < 0.01, n.s. no statistical significance. (**B**) The distribution of RNAs identified to be associated with telomere through CAPLOCUS followed by RNA-Seq. A total of 1923 telomere-associated RNAs were detected. (**C**) Enrichment of TERRA through counting sequencing reads containing repeated telomeric sequences in TERRA from CAPLOCUS followed by RNA-Seq. Only reads containing (TTAGGG)_4_ or (CCCTAA)_4_ telomeric motifs were counted. (**D**) Examples of RNAs associated with telomeres identified by CAPLOCUS followed by RNA-Seq.

Many non-coding RNAs play critical roles in transcriptional regulation and genome organization. For example, TERRA, as part of telomere architecture, has been implicated in the regulation of telomere length (45,46) and the formation of heterochromatin (47,48). Though various approaches have been developed to study RNA–chromatin interactions (49–51), these methods are limited to a specific RNA, and are not capable of identifying all chromatin-associated RNAs at a specific genomic locus. To examine whether CAPLOCUS can be used to identify chromatin-associated RNAs at a specific genomic locus, we tested the capture of RNAs in the telomere region using CAPLOCUS as a proof of concept. Reverse transcription followed by qRT-PCR showed a significant enrichment (4.22-fold) of TERRA in samples transfected with sgTelomere compared to control samples expressing sgGal4, while the negative control genes showed no significant enrichment (Figure 4A). To further investigate the RNAs enriched in the telomere region, we carried out RNA-Seq with the samples enriched from sgTelomere and sgGAL4. A final list of 1923 telomere-associated non-coding RNAs were identified (fold enrichment ≥ 3), with pseudogene, long intergenic non-coding RNA (IincRNA) and antisense as the largest components (Figure 4B). Consistent with the result generated from qPCR, counting sequencing reads containing (TTAGGG)_4_ or (CCCTAA)_4_ motifs (repeated sequences in TERRA), we observed a special enrichment of TERRA in sgTelomere compared to sgGAL4 control (Figure 4C). In addition, several known telomere-binding RNAs were also detected, such as RNA component of mitochondrial RNA processing endoribonuclease (RMRP) (52) (Supplementary Figure S6A) and small Cajal body-specific RNAs (scaRNAs) (53). Besides, several H/ACA small nucleolar RNA (snoRNA), which associated with a component of the telomerase complex—the nucleolar protein dyskerin (54,55), were also specially enriched in sgTelomere. These results suggest that CAPLOCUS is capable of identifying chromatin-associated RNAs at a specific genomic locus.

While no widely adopted approach is available to identify local interacting RNAs for a specific genomic locus, we compared our result to a method called MARGI (26), recently developed to massively reveal all potential chromatin-associated RNAs and their respective genomic-binding sites by proximity ligation. A total of 5441 non-coding RNAs potentially interacting with the telomeric region were found using pxMARGI, and a similar distribution of the RNA components was observed compared to that of CAPLOCUS (Figure 4B and Supplementary Figure S6B). We proceeded to compare the RNAs retrieved by CAPLOCUS and pxMARGI, and found that 12.6% of the RNAs identified by CAPLOCUS overlapped with those identified by pxMARGI (Supplementary Figure S6C). The difference between these two approaches could be explained in part by the nature of CAPLOCUS in only identifying the transient proximal interactions. Besides, CAPLOCUS utilized a mild (0.2%) formaldehyde treatment compared to pxMARGI (1%), which maintained the interactions close to the native state. Taken together, we believe that CAPLOCUS is an attractive platform in identifying chromatin-associated RNAs at a given locus.

### Identification of telomere-associated proteins by CAPLOCUS-MS

**Figure 5.**
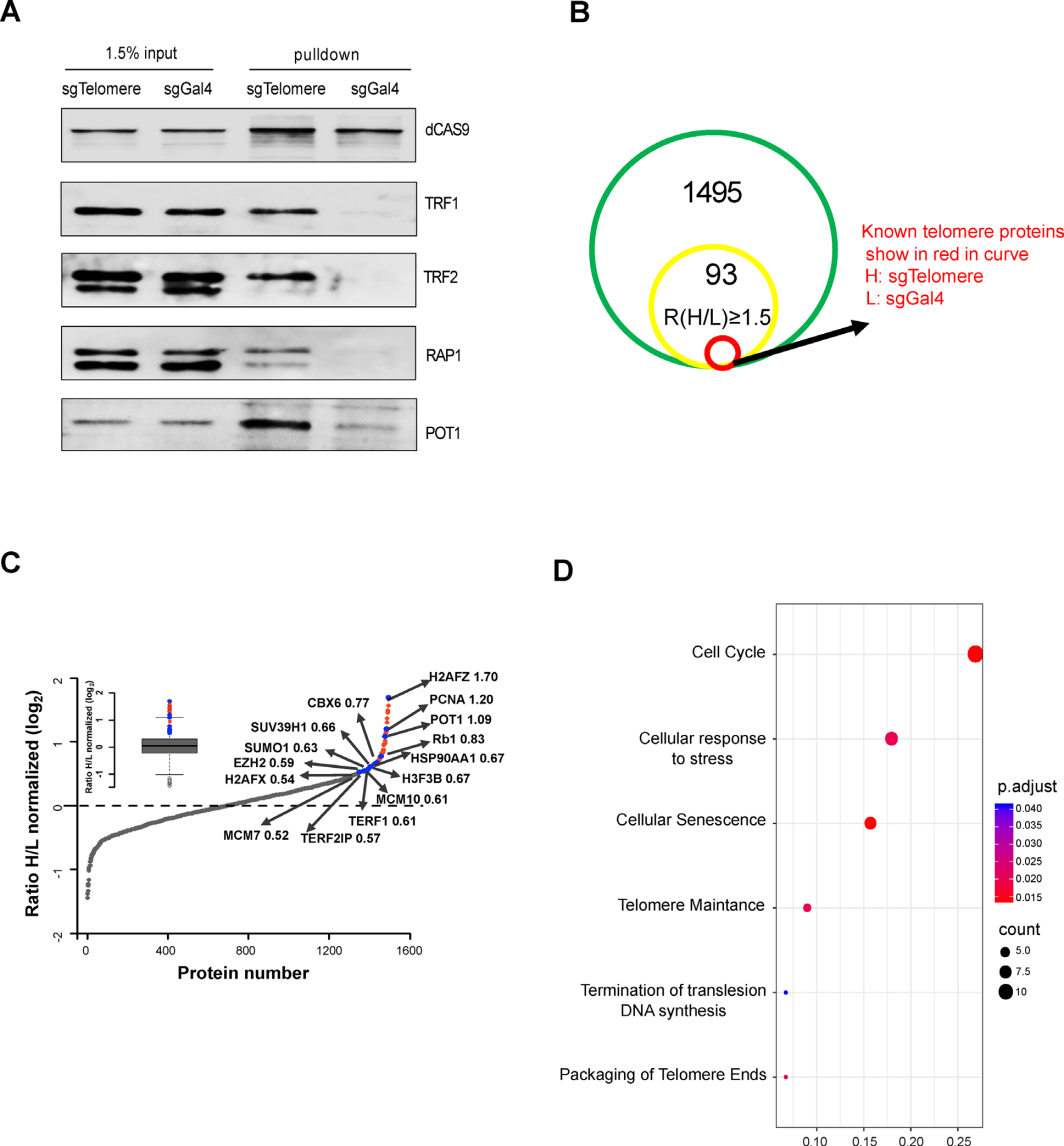
CAPLOCUS efficiently identify the proteins in telomeres. (**A**) Western blot shows enrichment of several known shelterin subunits of telomere in sgTelomere-expressing HEK293T cells, but not in the negative. (**B**) The number of proteins identified by CAPLOCUS followed by MS. The green circle represents the number of total identified proteins (non-specific proteins were removed as described in ‘Materials and Methods’ section) and the yellow one represents the potential telomere-associated proteins with normalized *H/L* ratio ≥ 1.5. The red circle represents known telomere-associated proteins. See also Supplementary Table S2. (**C**) The distribution of normalized *H/L* ratio from SILAC-MS. Small panel in the top left: a boxplot to illustrate the distribution of normalized log_2_(Ratio *H/L*) values of all identified proteins. 93 proteins (indicated as blue and red dots) were identified by CAPLOCUS with normalized *H/L* ratio ≥ 1.5. Data points representing the known telomere-associated proteins are highlighted in blue, and all the other proteins with *H/L* ratio ≥ 1.5 are indicated in red. The normalized log_2_(Ratio *H/L*) values of each known telomere-associated proteins are also included. H2AFX, MCM7 and TERF2IP are known telomere-associated proteins with normalized *H/L* ratio close to 1.5. (**D**) Functional profiles for gene clusters with a focus on Reactome pathway for potential telomere-associated proteins with normalized *H/L* ratio ≥ 1.5.

To evaluate whether CAPLOCUS is suitable for identifying chromatin-associated proteins in a specific genome region in their native state, we tested the CAPLOCUS approach in a well-studied telomere region. A total of 4 × 10^7^ cells was collected for CAPLOCUS by proximity biotin labeling and streptavidin-affinity purification. The purified samples were resolved by SDS-PAGE followed by western blotting. Known telomere shelterin subunits TRF1, TRF2, RAP1 and POT1 were highly enriched in samples transfected with sgTelomere, in contrast to samples expressing sgGal4, in which the shelterin subunits were not or slightly enriched (Figure 4A). Next, cultured cells were subjected to stable isotope labeling (SILAC) followed by CAPLOCUS purification and MS to identify specific enriched proteins in samples with telomere-targeted sgRNAs versus the samples with the sgGal4. A list of proteins known to reside in cytoplasm and should not be biotin-labeled were chose as false positives (Supplementary Table S2). We surveyed the distribution of normalized *H/L* ratio of these proteins, and observed that majority of them (83%) have a *H/L* ratio <1.5, thus a *H/L* ratio ≥1.5 was considered as a cutoff (Supplementary Figure S7A). We identified a total of 93 proteins (Figure 4B and Supplementary Figure S7B), including many previously reported telomere-associated ones (10,56,57), such as the shelterin subunits POT1 and TERF1 (Figure 5C). Functional relevance of these identified proteins was evaluated and they were found to be significantly enriched in the pathways of cell cycle, cellular responses to stress, telomere maintenance and packaging of telomere ends (Figure 4D).

Telomere-associated proteins have been identified previously by PICh (proteomics of isolated chromatin segments) (10) and BioID (proximity-dependent biotinylation) (58). When our manuscript was under revision, two similar methods called C-BERST (dCas9-APEX2 biotinylation at genomic elements by restricted spatial tagging) (59) and GLoPro (genomic locus proteomics) (60) were reported separately, both of which identify chromatin-associated proteins at a specific chromatin locus through the combination of dCas9 and APEX2. Similar to what we did, telomere-associated proteins were also investigated by C-BERST in human U2OS cells. Comparing telomere-associated proteins identified through these four approaches, we found 19 proteins identified by CAPLOCUS were also detected by at least one of the other methods (Supplementary Figure S8 and Supplementary Table S2). For the remaining proteins only identified by CAPLOCUS, five of them are known telomere-associated ones (Supplementary Figure S8) (61–65), including three proteins (Rb1 (61), SUV39H1 (63), EZH2 (65)) previously reported to directly bind the TERRA or its promoter in the establishment of telomeric heterochromatin. Taken together, these results indicate that CAPLOCUS followed by MS can be successfully used to identify nearby chromatin-associated proteins for a given locus.

## DISCUSSION

In this study, we developed CAPLOCUS based on a modified CRISPR system to isolate *in vivo* chromatin components in a specific genomic region. We showed that CAPLOCUS could efficiently isolate repetitive regions (telomere, C13) as well as two single-copy loci (C11 and 3′HS1) with high resolution. Combined with high-throughput sequencing, CAPLOCUS is also capable of identifying long-range DNA interactions in a single-copy locus. CAPLOCUS is also capable of identifying telomere-associated RNAs after RNA-Seq of enriched samples. Moreover, this method allows the enrichment of proteins associated with chromatin in their native states, and we were able to successfully identify telomere-binding proteins via western blot analysis and CAPLOCUS-MS. Hence, the CAPLOCUS method provides a novel approach for investigating locus-specific interactions without prior understanding of the target loci.

To optimize the CRISPR-based system, we also tested another type of CRISPR system (66) with different promoter and nuclear localization signal (NLS) (Supplementary Figure S9A). Although it had good resolution, this system showed lower enrichment of C11 compared with the system used above (Supplementary Figure S9B). We also noticed that the low expression levels of APEX2 were vital for the successful enrichment of CAPLOCUS (Supplementary Figure S9C), which could be achieved either with low amount of APEX2 transformants or by utilizing stable cells with low APEX2 expression (15).

Recently, Liu *et al.* developed a novel method called CAPTURE (CRISPR affinity purification in situ of regulatory elements) (67) to study long-range DNA interactions and chromatin-associated proteins. Similar to CAPLOCUS, CAPTURE utilizes high-affinity streptavidin purification to efficiently capture protein–DNA complexes. However, CAPLOCUS utilizes an engineered ascorbate peroxidase APEX2 to directly label nearby proteins with biotin, enabling the isolation of proteins associated with chromatin in their native states for a specific region, whereas CAPTURE uses the biotin ligase BirA to label dCas9 with biotin and requires crosslinking to purify the locus-specific proteins. Moreover, due to the faster labeling kinetics of APEX2, CAPLOCUS has the potential to study locus-specific interactions in a limited period of time, which may not be achievable with CAPTURE. During the revision of this manuscript, C-BERST (59) and GLoPro (60) were reported to utilize dCas9 and APEX2 to investigate the proteomes near defined genomic loci, which is similar to CAPLOCUS. Although the identified proteins were partially overlapped between C-BERST and CAPLOCUS, which may attribute to the different cell lines used in the two methods (U2OS for C-BERST and HEK293T for CAPLOCUS), a similar discovery rate of reported telomere-associated proteins were observed, with 30 out of 143 (∼21%) proteins for C-BERST and 24 out of 93 (∼25.8%) proteins for CAPLOCUS. Though both C-BERST and GLoPro mentioned the possibility of discovering the chromatin-associated RNAs or long-range DNA elements, neither of them provides detailed evidence. Hence, to our knowledge, CAPLOCUS is the first methodology available which can be used to generate a comprehensive profile of all interacting DNAs, RNAs and proteins at any specific genomic locus, which will be extremely useful in deciphering the roles of these molecules in chromatin architecture.

For long-range DNA interaction analysis, we compared CAPLOCUS with the classical 4C-Seq method. While both methods are effective in identifying long-range DNA interactions, differences on these two approaches need to be noticed. The 4C method uses enzyme-based step to fragment the chromatin DNA, and the result depends on the enzyme distribution and efficiency of proximity ligation. In contrast, CAPLOCUS utilizes sonication to randomly fragment the chromatin DNA. Although this may generate less distal intra-chromosomal interactions as previously reported (68), it provides a higher resolution of the target site. Besides, unlike 4C, CAPLOCUS enables the snapshot of the interacting DNAs at a given locus through temporal labeling, together with the utilization of robust streptavidin–biotin interaction, which can endure much more rigorous handling, CAPLOCUS can achieve high specificity with limited contaminations. This can, in part, explain why we identified much less interactions (150 interactions for CAPLOCUS; 448 interactions for 4C) with a relatively high accuracy (46%) and low sensitivity (15.4%).

Despite the advantages shown above, CAPLOCUS does have some limitations. First, the radius of the biotin-phenoxyl radical is not a fixed value and may detach from APEX2 several nanometers away (15); in other words, proteins that are several nanometers away from the target region may still be biotinylated. To achieve high-specificity enrichment of the target region, control cells should be treated in parallel and analyzed together. Second, because APEX2 directly labels the proteins in the proximity of the target region with biotin, components that directly and indirectly interact with the target region may both be detected. Third, because proteins associated with a specific locus may be very limited, a large number of cells may be needed for protein identification. In our experiment, CAPLOCUS efficiently identified four known shelterin components that interact with the telomeric region, as verified by western blot analysis. However, it failed to identify many known telomere-binding proteins, which may be improved by the development of deep efficient peptide sequencing and sensitive quantitative proteomics (69–71). Finally, APEX2 has fast-labeling kinetics, which is beneficial for a snapshot of the target but can also lead to missed components because chromatin change dynamically. This can result in an incomplete understanding of the target region.

The investigation of chromatin organization has attracted the attention of global scientists. Recently, the 4D Nucleome Project (72) was proposed to develop and apply approaches to gain a deeper understanding of nuclear organization and function. In addition, genome-wide association studies have shown that the majority of disease-associated variants are located in regulatory, non-coding regions (73), indicating the importance of gene regulation by long-range chromatin interactions. CAPLOCUS provides a novel approach for studying interacting molecules in a specific genomic region with a spatial-temporal resolution, which may facilitate our understanding of the 3D genome organization and its dynamic changes in both normal and disease states.

## DATA AVAILABILITY

Sequencing data have been deposited in the Gene Expression Omnibus, GEO: GSE114133.

## Supplementary Material

Supplementary Data
