# Supplementary material for: Determination of local chromatin interactions using a combined CRISPR and peroxidase APEX2 system
Source: Nucleic Acids Res. 2019 Feb 26;47(9):e52. doi: 10.1093/nar/gkz134 (PMC6511869; doi:10.1093/nar/gkz134)
Supplement: Supplementary Data [file gkz134_supplemental_files.zip › Supplement Figure.pdf]

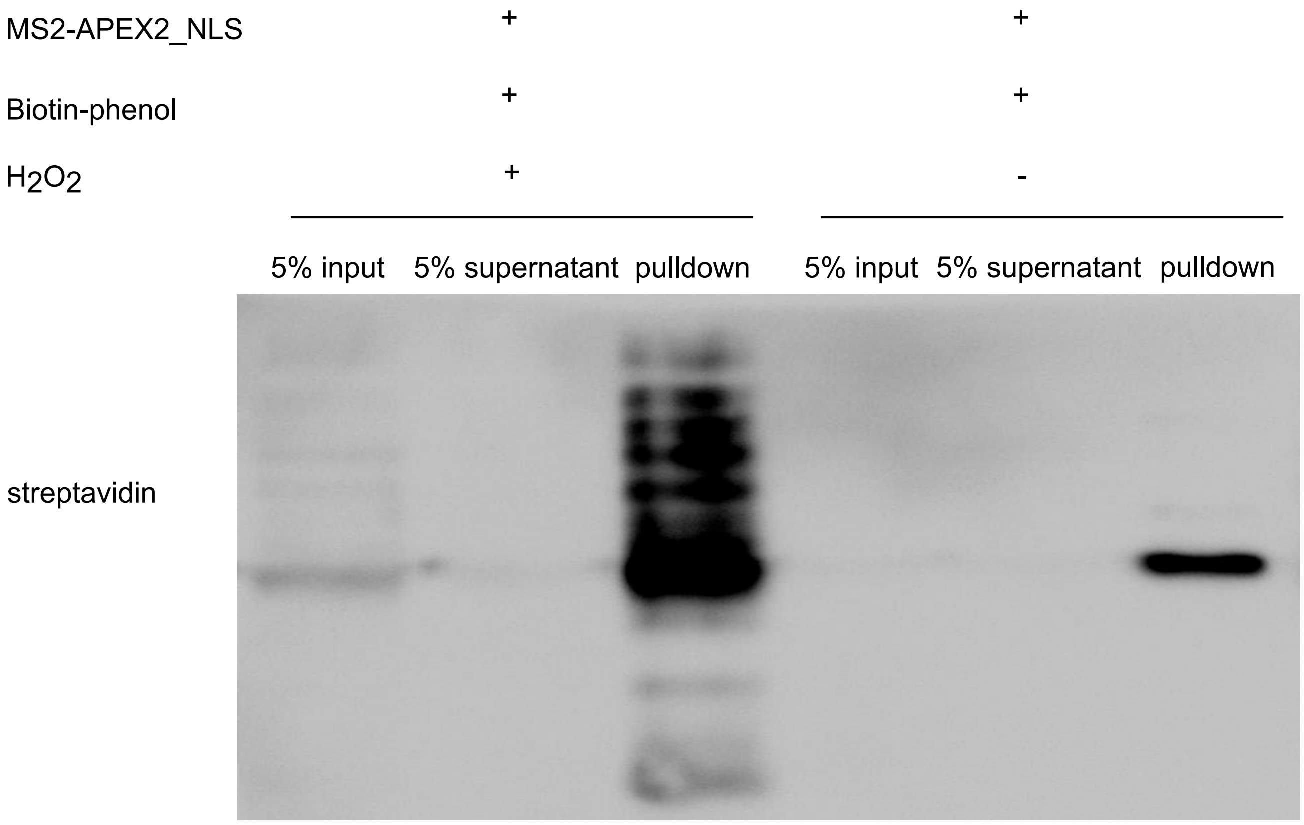

Figure S1. APEX2-mediated biotinylation of endogenous proteins by streptavidin blotting. HEK293T cells were transfected with MS2-APEX2_NLS, cells were biotin-labeled, lysed, affinity purification with streptavidin and separated on SDS-PAGE. Biotin-labeled proteins were detected by blotting with streptavidin-HRP. Negative controls in which H_2O_2 was omitted was shown. In negative control cells, only endogenously biotinylated proteins are detected, however, the biotinylated proteins by APEX2 labeling are dramatically enriched in cells transfected with MS2-APEX2_NLS.

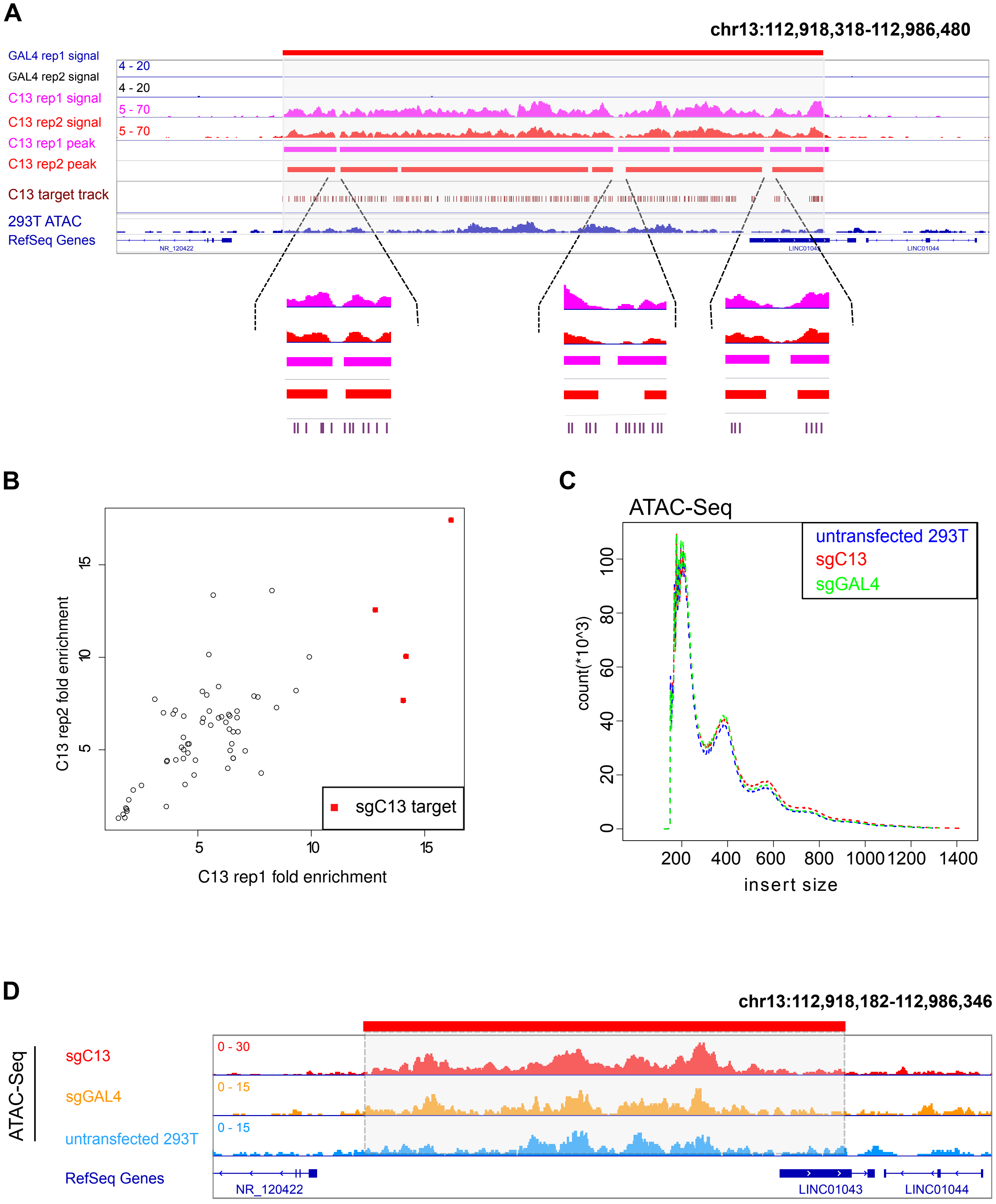
A
chr13:112,918,318-112,986,480
GAL4 rep1 signal
GAL4 rep2 signal
C13 rep1 signal
C13 rep2 signal
C13 rep1 peak
C13 rep2 peak
C13 target track
293T ATAC
RefSeq Genes
4 - 20
4 - 20
5 - 70
5 - 70
B
C13 rep2 fold enrichment
C13 rep1 fold enrichment
sgC13 target
5
10
15
C
ATAC-Seq
untransfected 293T
sgC13
sgGAL4
count(*10^3)
insert size
0
20
40
60
80
100
200
400
600
800
1000
1200
1400
D
chr13:112,918,182-112,986,346
ATAC-Seq
sgC13
sgGAL4
untransfected 293T
RefSeq Genes
0 - 30
0 - 15
0 - 15
NR_120422
LINC01043
LINC01044

Figure S2. Sensitivity and specificity of CAPLOCUS on C13 (chr13: 112,930,173-112,968,847) region.

(A) High specificity of CAPLOCUS on the target C13 region. Replicate experiments (rep1 and rep2) are shown, cells expressing sgGAL4 were analyzed as negative controls. The region encompassing the C13 locus is indicated in red bars on the top. The sgC13 targeting locations are indicated in brown. Three regions without identified peaks in both experiments are enlarged, no dCas9/sgC13 binding sites were found in these regions.

(B) Genome-wide analysis of chromatin regions enriched by CAPLOCUS on the C13 target. Replicate experiments (rep1 and rep2) are shown. Called peaks for the target C13 region are highlighted in red.

(C) Nucleosome positioning evaluated by ATAC-Seq in untransfected HEK293T cells (blue), or cells with the CAPLOCUS system targeting C13 (red) or Gal4 (green). The comparison of insert fragment size is shown.

(D) The comparison of local chromatin accessibility on the target region (C13) for untransfected HEK293T cells, or cells with the CAPLOCUS system targeting C13 or Gal4 by ATAC-Seq data. The target region is indicated in red on the top.

A

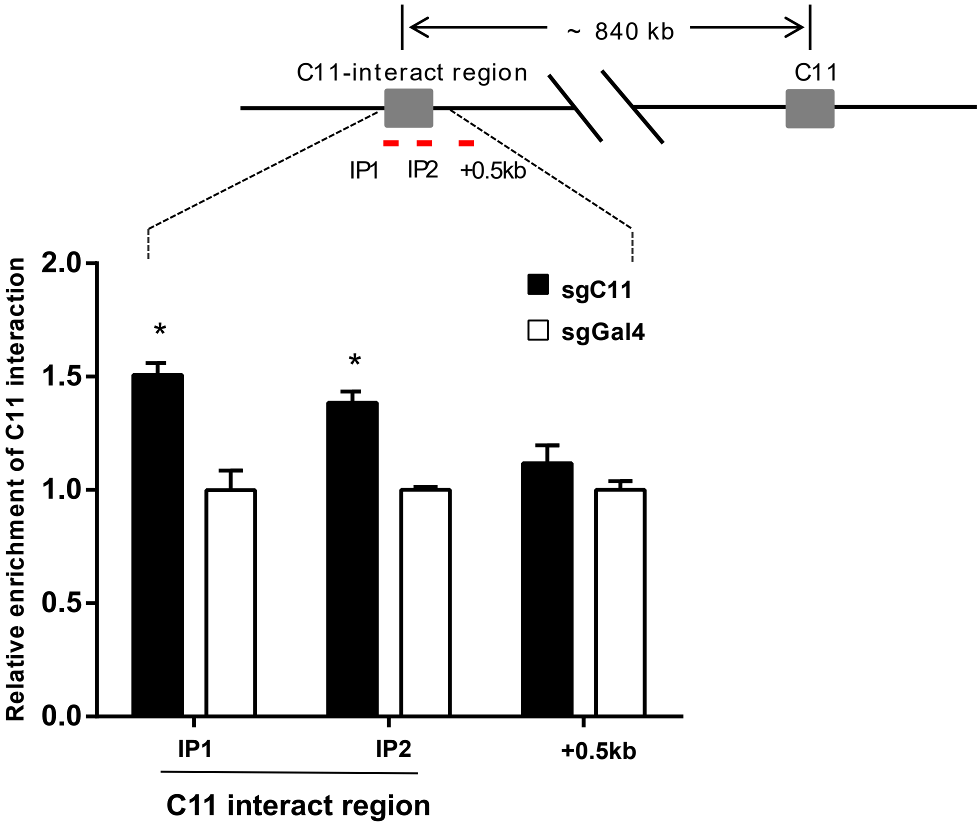

B

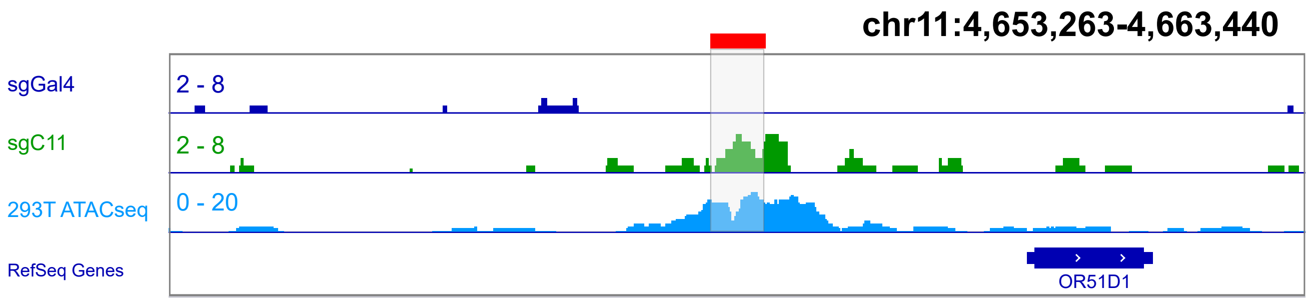

Figure S3. A region with known long-range interaction with C11 was detected.

(A) qPCR analysis shows significant enrichment of the known region (chr11:4,658,147-4,658,496) interacting with C11. Regions amplified by qPCR are indicated in red. The relative enrichment was calculated as described in Figure 2B. Error bars are mean ± SEM of three experiments and analyzed by a two-sided t test. *p< 0.05.

(B) The interacting region is enriched with next generation sequencing after CAPLOUCS. The locus of the interacting region is indicated in red.

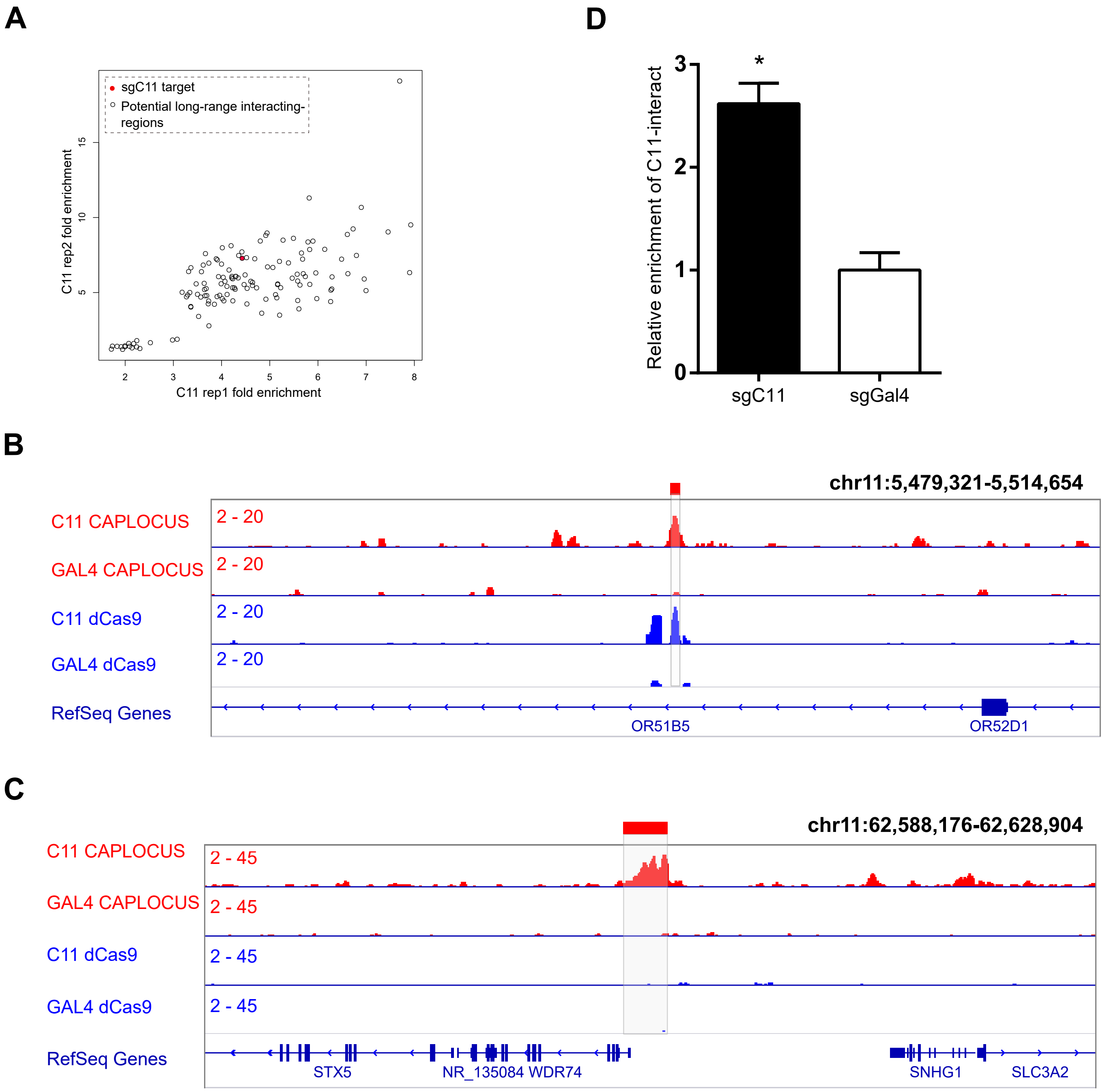

Fiugre S4. Genome-wide enrichment and specificity of CAPLOCUS on C11 region compared with dCas9 ChIP-seq.

(A) Genome-wide analysis of chromatin regions enriched by CAPLOCUS on the C11 target. Replicate experiments are shown. Data points for the C11 target region is shown as red.

(B) The C11 target region was successfully enriched in both CAPLOCUS and dCas9 ChIP-seq. C11 sgRNA targeting locations are indicated in red bar on the top. Cells expressing sgGal4 were analyzed as negative controls.

(C) Example of a long-range interaction (chr11: 62607280 - 6609275) of C11 identifyed by CAPLOCUS. CAPLOCUS and dcas9 ChIP-seq signals on the potential interacting region (chr11: 62607280 - 6609275) are shown. dCas9 ChIP-seq shows no enrichment of this region in cells expressing sgC11. Cells expressing sgGal4 were analyzed as negative controls.

(D) qPCR analysis shows significant enrichment of the identified long-range interaction region indicated in C. The relative enrichment was calculated as described in Figure 2B. Error bars are mean ± SEM of three experiments and analyzed by a two-sided t test , $*p < 0.05$.

A

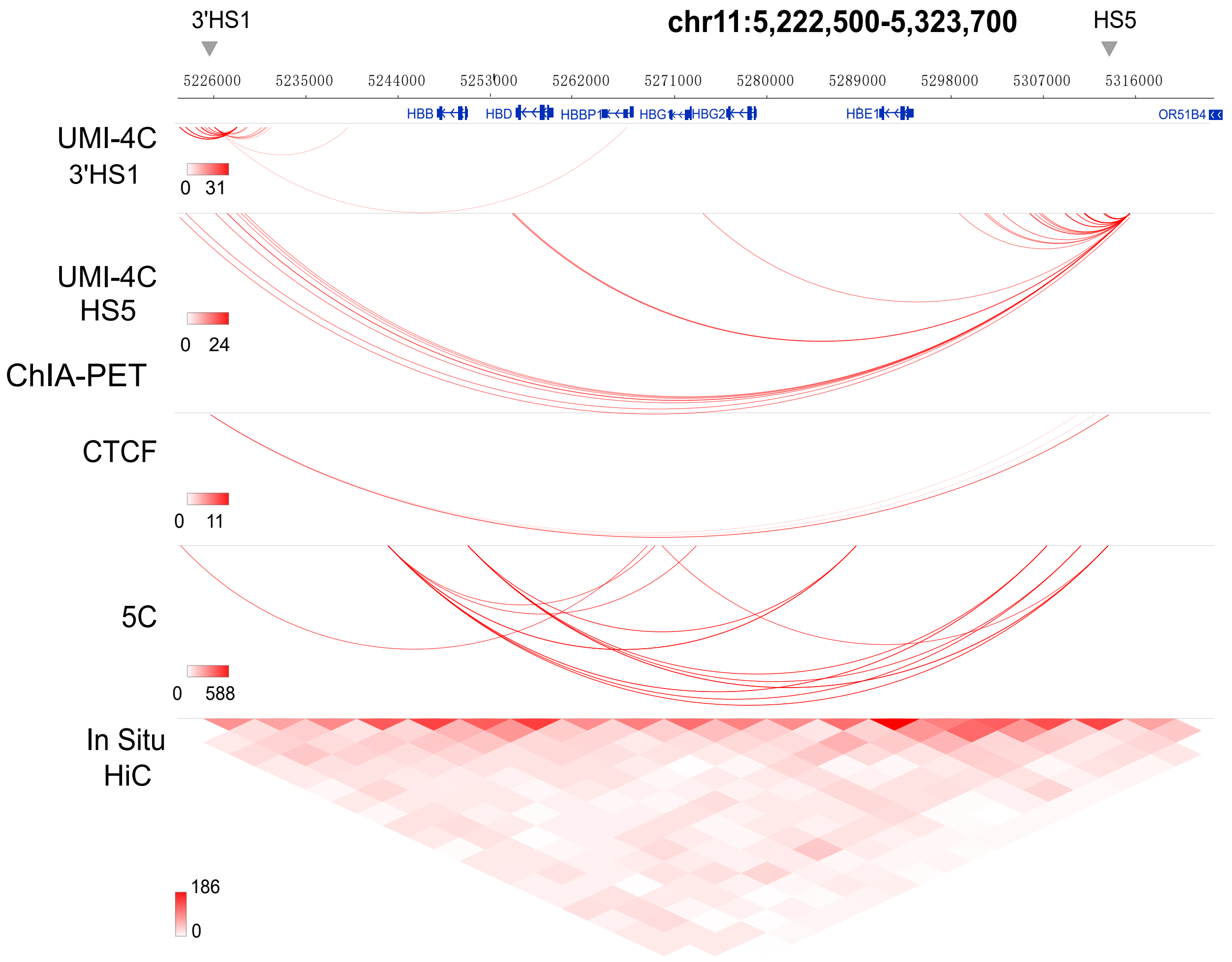
3'HS1
chr11:5,222,500-5,323,700
HS5
5226000
5235000
5244000
5253000
5262000
5271000
5280000
5289000
5298000
5307000
5316000
HBB
HBD
HBBP1
HBG1
HBG2
HBE1
OR51B4
UMI-4C
3'HS1
0 31
UMI-4C
HS5
0 24
ChIA-PET
CTCF
0 11
5C
0 588
In Situ
HiC
186
0

B

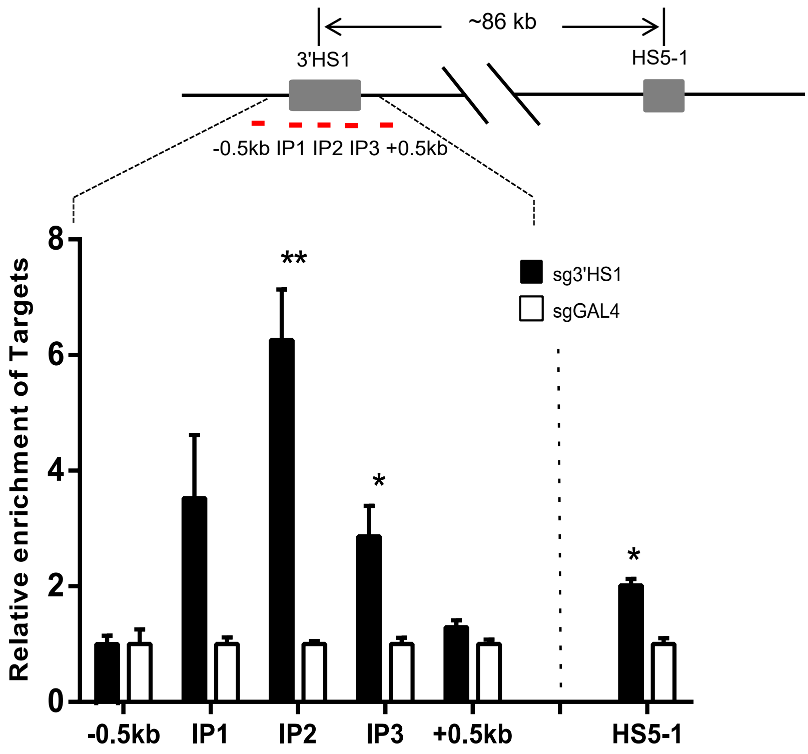
~86 kb
3'HS1
HS5-1
-0.5kb IP1 IP2 IP3 +0.5kb
Relative enrichment of Targets
0
2
4
6
8
**
*
*
sg3'HS1
sgGAL4
-0.5kb
IP1
IP2
IP3
+0.5kb
HS5-1

Figure S5. The comparison of CAPLOCUS with other methods to characterize the long-range chromatin interactions on a single-copy locus (3'HS1, chr11:5,226,031-5,226,405).

(A) Published results from UMI-4C using HS5 or 3'HS1 as baits, CTCF ChIA-PET (ENCODE Project Consortium, Nature. 2012), 5C (Naumova *et al.*, Science. 2013) and *in situ* Hi-C (Rao *et al.*, Cell. 2014) on the well-characterized β-globin locus in K562 cells are shown.

(B) The significant enrichment of the 3'HS1 target region as well as its interacting region (HS5, chr11:5,312,339-5,312,938) by the CAPLOCUS system. qPCR amplicon regions are indicated in red. The relative enrichment was calculated as described in Figure 2B. Error bars are mean ± SEM of three experiments and analyzed by a two-sided t test , $*p < 0.05$.

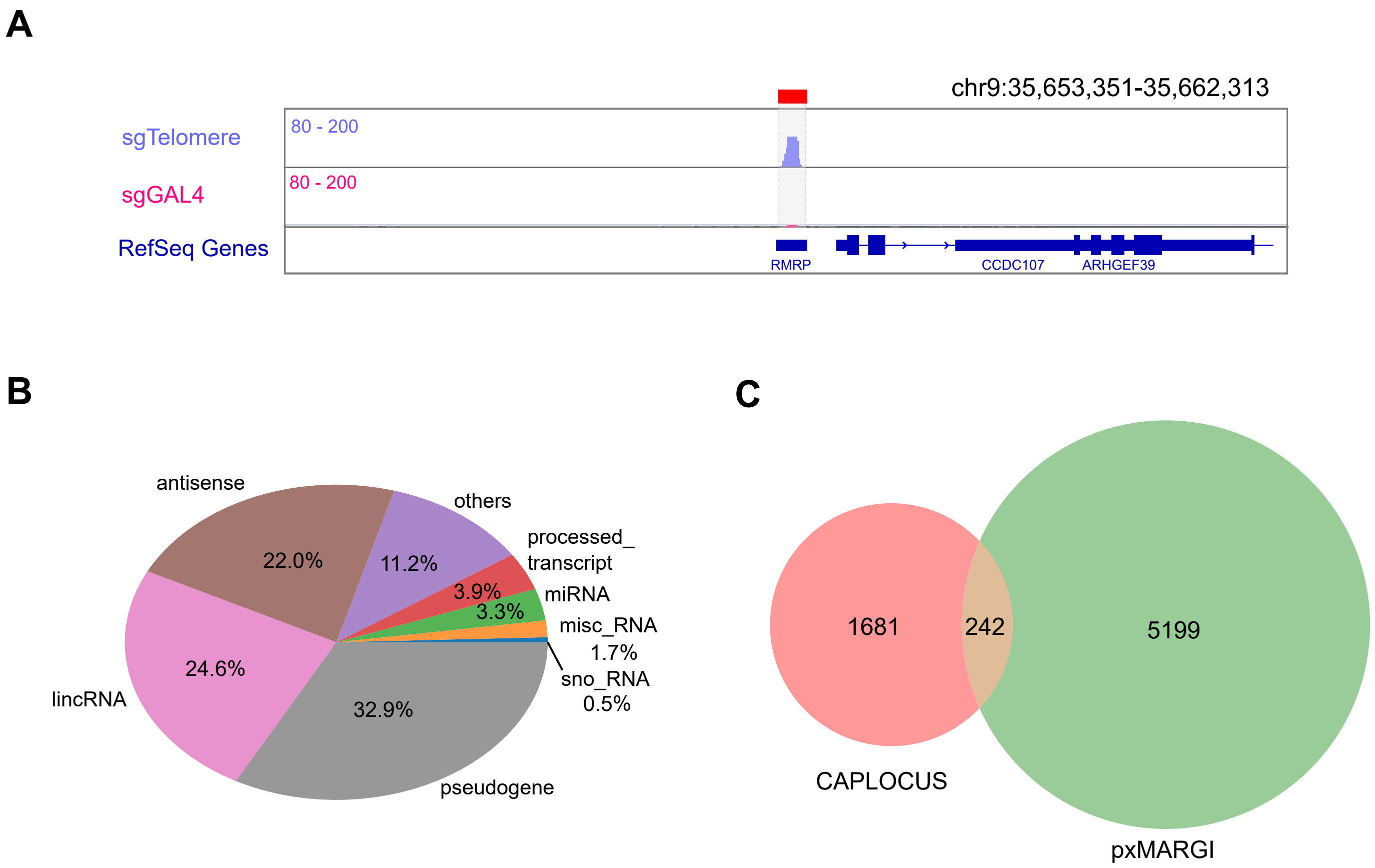

Fiugre S6. CAPLOCUS-RNA-seq identified known and new telomere-associated RNAs.

(A) A known telomere-associated RNA named RMRP was detected by CAPLOCUS-RNA-seq. Reads coverage of RMRP transcript in sgTelomere and sgGAL4 samples are shown.

(B) Classification of RNAs associated with telomeric region retrieved from pxMARGI (Sridhar B *et al.*, Curr Biol. 2017) in HEK293T cells. A total of 5441 telomere-associated RNAs were obtained. The distribution of the RNA components was similar with that of CAPLOCUS-RNA-seq (Figure 4B).

(C) Venn diagram of telomere-associated RNAs identified by CAPLOCUS-RNA-seq and pxMARGI.

A

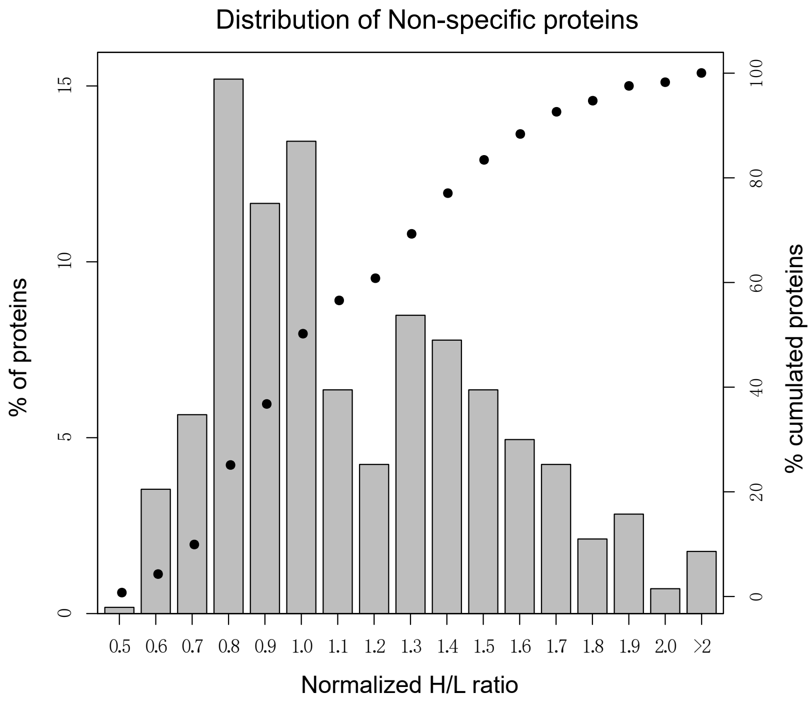

B

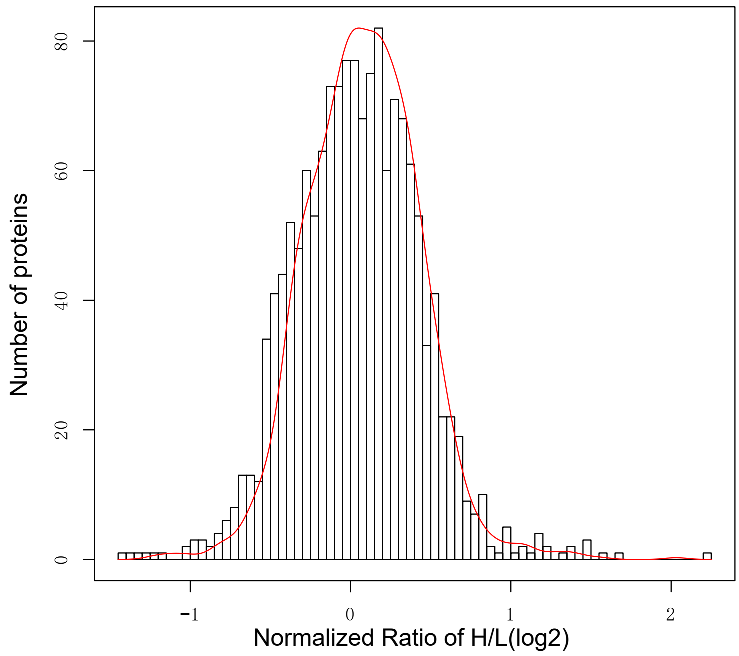

Figure S7. The distribution of proteins in CAPLOCUS-MS across H/L ratio.
(A) The distribution of non-specific proteins across H/L ratio. The bars represent the percentage (%) of non-specific proteins (left y axis) in each category, black circles represent the cumulative % of non specific proteins (right y axis).
(B) The distribution of all proteins (non-specific excluded) across normalized ratio of log2(H/L).

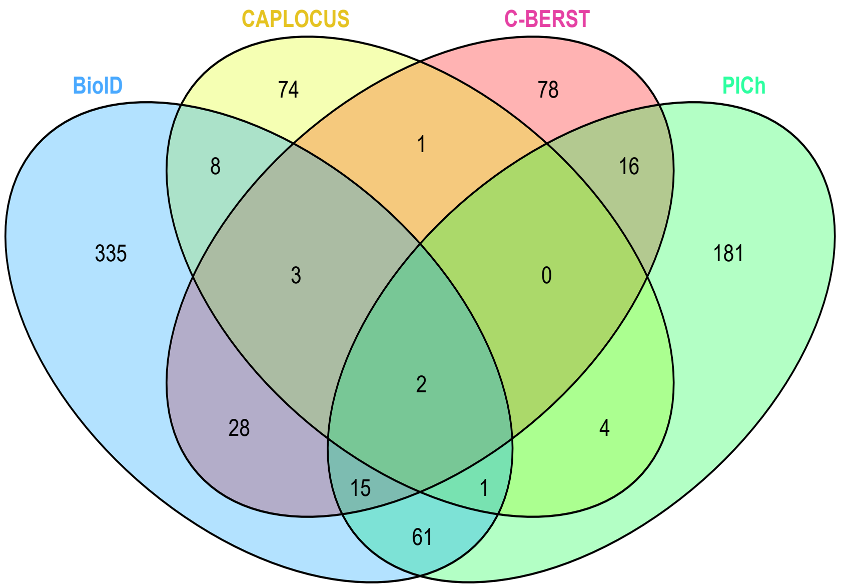

Examples of proteins shared between CAPLOCUS and other methods:

H2AZ, ATAD5, PCNA, UQCRC1, POT1, USP36, WHSC1, TERF1, MCM10

Known telomere-associated proteins specially identified by CAPLOCUS:

Rb1, HSP90AA1, SUV39H1, SUMO1, EZH2

Figure S8. Comparison of CAPLOCUS telomeric dataset with data from other approaches. Venn diagram of proteins identified by CAPLOCUS, PICh (Déjardin J *et al.*, Cell. 2009), BioID (Garcia-Exposito L *et al.*, Cell Rep. 2016.) and C-BERST (Gao XD, *et al.*, Nat Methods. 2018). Among the 93 proteins determined by CAPLOCUS, 19 proteins were also detected by at least one other method. Examples of the shared proteins are shown below the chart. Most of them are highly enriched in CAPLOCUS (log2(Ratio H/L normalized) $\geqslant$ 0.585). Known telomere-associated proteins specially detected by CAPLOCUS are shown. See also Table S2.

A

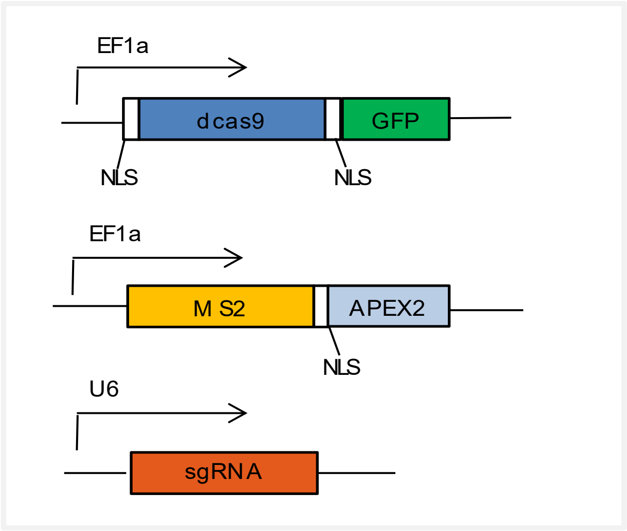
EF1a
dcas9
GFP
NLS
NLS
EF1a
MS2
APEX2
NLS
U6
sgRNA

B

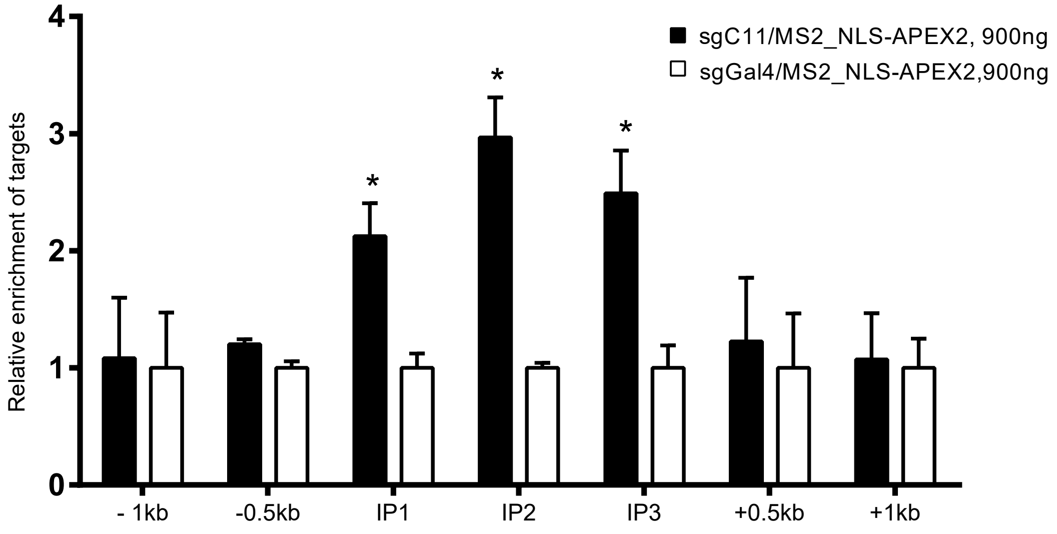
sgC11/MS2_NLS-APEX2, 900ng
sgGal4/MS2_NLS-APEX2,900ng
Relative enrichment of targets
0
1
2
3
4
*
*
*
- 1kb
-0.5kb
IP1
IP2
IP3
+0.5kb
+1kb

C

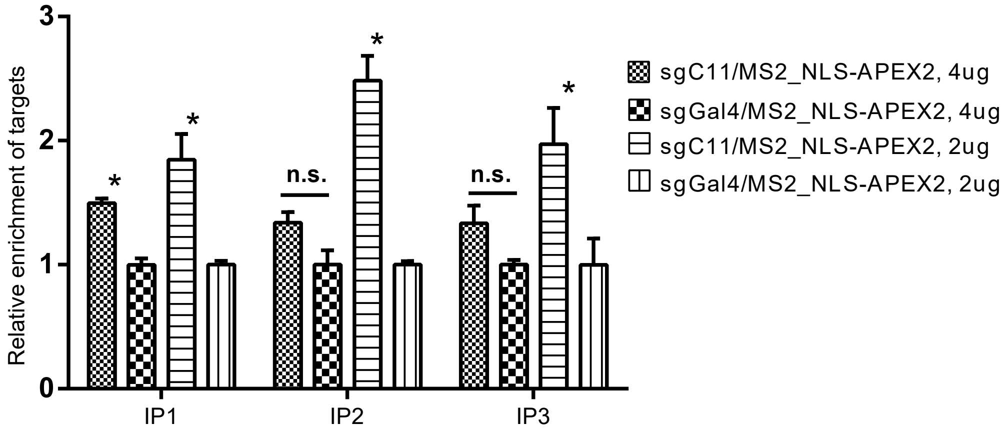
sgC11/MS2_NLS-APEX2, 4ug
sgGal4/MS2_NLS-APEX2, 4ug
sgC11/MS2_NLS-APEX2, 2ug
sgGal4/MS2_NLS-APEX2, 2ug
Relative enrichment of targets
0
1
2
3
*
*
n.s.
*
n.s.
*
IP1
IP2
IP3

Figure S9. CAPLOCUS of C11 with another plasmid system.

(A) The three components of the system: EF1a-NLS_dCas9_NLS (derived from Addgene 61422), MS2-NLS_APEX2 (derived from Addgene 61423), and target-specific sgRNA (Addgene 61424).

(B) The C11 target is significantly enriched using this system. However, the fold enrichment is lower compared with TO-NLS_dCas9_NLS/ MS2-APEX2_NLS/ sgRNA. qPCR amplicon regions are indicated in Figure 3A. The relative enrichment was calculated as described in Figure 2B. Error bars are mean ± SEM of three experiments and analyzed by a two-sided t test, *$p < 0.05$.

(C)The amount of APEX2 plasmid transformed in CAPLOCUS is vital for the successful enrichment of the target. C11 is enriched using CAPLOCUS with different amounts of MS2_NLS-APEX2. qPCR analysis shows an increased fold enrichment of the C11 target region when the amount of MS2_NLS - APEX2 decreased. The relative enrichment was calculated as described in Figure 2B. Error bars are mean ± SEM of three experiments and analyzed by a two-sided t test, *$p < 0.05$.
